# Development of an Injectable, ECM-Derivative Embolic for the Treatment of Cerebral Saccular Aneurysms

**DOI:** 10.1021/acs.biomac.4c00321

**Published:** 2024-07-13

**Authors:** Seungil Kim, Kamil W. Nowicki, Keishi Kohyama, Aditya Mittal, Sangho Ye, Kai Wang, Taro Fujii, Shivbaskar Rajesh, Catherine Cao, Rohit Mantena, Marianna Barbuto, Youngmee Jung, Bradley A. Gross, Robert M. Friedlander, William R. Wagner

**Affiliations:** † Department of Bioengineering, University of Pittsburgh, Pittsburgh, Pennsylvania 15261, United States; ‡ Department of Surgery, University of Pittsburgh, Pittsburgh, Pennsylvania 15261, United States; § McGowan Institute for Regenerative Medicine, University of Pittsburgh, Pittsburgh, Pennsylvania 15261, United States; ∥ Department of Neurosurgery, School of Medicine, University of Pittsburgh, Pittsburgh, Pennsylvania 15261, United States; ⊥ Department of Neurosurgery, School of Medicine, Yale, New Haven, Connecticut 06520, United States; # Discovery Center for Musculoskeletal Recovery, Schoen Adams Research Institute at Spaulding, Charlestown, Massachusetts 02115, United States; ∇ Department of Physical Medicine and Rehabilitation, Harvard Medical School, Boston, Massachusetts 02115, United States; ○ Division of Experimental Pathology, Department of Pathology, University of Pittsburgh School of Medicine, Pittsburgh, Pennsylvania 15213, United States; ◆ Ri.MED Foundation, Cardiac Tissue Engineering Laboratory, Ri.MED Foundation, Palermo 90133, Italy; ¶ Department of Biological, Chemical and Pharmaceutical Sciences and Technologies (STEBICEF), University of Palermo, Palermo 90133, Italy; k Center for Biomaterials, Biomedical Research Institute, Korea Institute of Science and Technology (KIST), Seoul 130-650, Republic of Korea; l School of Electrical and Electronic Engineering, YU-KIST Institute, Yonsei University, Seoul 130-650 Republic of Korea

## Abstract

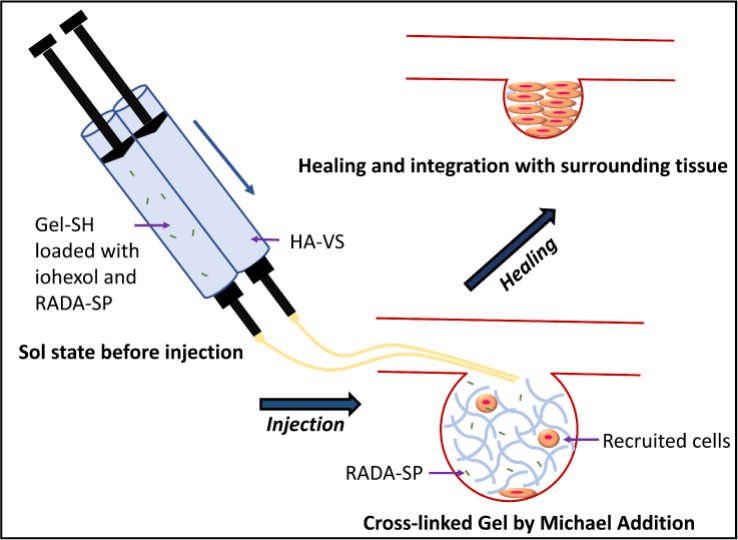

Cerebral aneurysms are a source of neurological morbidity and mortality, most often as a result of rupture. The most common approach for treating aneurysms involves endovascular embolization using nonbiodegradable medical devices, such as platinum coils. However, the need for retreatment due to the recanalization of coil-treated aneurysms highlights the importance of exploring alternative solutions. In this study, we propose an injectable extracellular matrix-derived embolic formed in situ by Michael addition of gelatin-thiol (Gel-SH) and hyaluronic acid vinyl sulfone (HA-VS) that may be delivered with a therapeutic agent (here, RADA-SP) to fill and remodel aneurysmal tissue without leaving behind permanent foreign bodies. The injectable embolic material demonstrated rapid gelation under physiological conditions, forming a highly porous structure and allowing for cellular infiltration. The injectable embolic exhibited thrombogenic behavior in vitro that was comparable to that of alginate injectables. Furthermore, in vivo studies in a murine carotid aneurysm model demonstrated the successful embolization of a saccular aneurysm and extensive cellular infiltration both with and without RADA-SP at 3 weeks, with some evidence of increased vascular or fibrosis markers with RADA-SP incorporation. The results indicate that the developed embolic has inherent potential for acutely filling cerebrovascular aneurysms and encouraging the cellular infiltration that would be necessary for stable, chronic remodeling.

## 1 Introduction

Cerebral aneurysms are thought to develop at points of hemodynamic stress in intracranial arteries and are characterized by their bulging, saccular structure.^1,2^ Cerebral aneurysms affect approximately 2–3% of the adult population, with each aneurysm carrying an average annual rupture risk of 1% (although certain aneurysms can have a rupture risk as high as 50%) and a mortality rate of 50% when rupture occurs.^3^

Recently, the use of nondegradable platinum coils to embolize cerebral aneurysms has gained widespread acceptance for their treatment.^4^ Additionally, flow-diverting-device (FDD) stents with high metal coverage and low porosity have been introduced. While FDD has greater rates of aneurysm obliteration than intrasaccular therapy, careful patient selection is necessary for typical side-wall aneurysms, and extended antiplatelet therapy is required.^5^

Despite its advantages, endovascular therapy using coiling is not without limitations and complications. These include well-known issues such as coil migration, coil compaction, and recanalization.^6^ Recanalization has been observed in approximately 17% of patients who undergo bare coiling for aneurysms, necessitating retreatment in 10.3% of cases.^7,8^ The occurrence of recanalization is more common in the cases of large, giant, or wide-neck aneurysms.

As an alternative to metallic coiling, researchers have investigated injectable embolic agents to fill vascular defects including arteriovenous malformations and aneurysms.^9^ These include Onyx, *n*-butyl cyanoacrylate, and Embogel.^5^ Here, the target site is filled by administering a controlled amount of the embolic agent to occlude the aneurysm. However, Onyx and *n*-butyl cyanoacrylate have limitations primarily as a result of uncontrolled reflux into the parent artery, creating a higher risk of stroke.^10^ They are thus not used in the treatment of typical saccular aneurysms. EmboGel is an alginate-based embolic biomaterial that does not require organic solvents and achieves solidification by the introduction of calcium chloride. Although EmboGel is biocompatible, the resulting solidified gel with calcium chloride may be soft and fragile, leading to the potential for rapid degradation and washout of the parent artery.

Recently, extensive research has been conducted on gelatin for the development of injectables aimed at treating hemorrhage. Gelatin has been explored as a shear-thinning,^11−15^ semisolid,^16,17^ and cross-linkable hydrogel.^18,19^ These studies have been motivated by the various characteristics of gelatin, such as its ability to trigger clot formation, low immunogenicity, and support for cell attachment and proliferation. Among these approaches, injectables composed of gelatin and nanoclay have shown promising results in hemorrhage control and embolization.^16,17^ However, it has been reported that silicate nanoplatelets can induce cytotoxicity through the generation of reactive oxygen species, damage to cell membrane interactions, and intracellular interaction mechanisms.^20^

In light of these concerns, the development of extracellular matrix (ECM)-derived cross-linkable hydrogels, such as those based on modified gelatin and hyaluronic acid, has garnered attention as injectable gels due to their cytocompatibility, biological functionality, and slow degradability relative to physically cross-linked gels.^18,19,21^ Recently, combined gelatin and hyaluronic acid hydrogels have been studied as injectable, cross-linking embolic agents.^19^ However, chemically cross-linkable and injectable ECM-mimicking gels of gelatin and hyaluronic acid have not been explored as a means to fill saccular aneurysms by embolization and to facilitate healing.

For achieving long-lasting exclusion and stabilization of the vessel wall without the placement of permanent foreign bodies, it would be ideal if an ECM-derived embolic was employed that initially facilitates thrombogenesis and subsequent cell attachment and retention, followed by gradual degradation and resorption. To meet those requirements, in this study, we developed a modified form of gelatin and hyaluronic acid to create an injectable and cross-linkable ECM-derived embolic agent. Our goal was to deliver the gel, with or without an additional therapeutic agent, to promote a healing response and facilitate tissue-in-growth to the aneurysm sac. As a model therapeutic agent, the self-assembling peptide (RADA) was conjugated with neuropeptide substance P (SP) (RADA-SP, Ac-RARADADARARADADAGGRPKPQQFFGLM-NH_2_). RADA-SP was selected due to its reported ability to enhance the recruitment of endogenous cells into implanted scaffolds and expedite the process of wound healing.^22^ Also, the effectiveness of RADA-SP in recruiting vascular smooth muscle cells (vSMCs) is well-proven in published papers.^22,23^ The addition of RADA-SP to the injectable embolic aimed to foster increased in-growth of vSMCs to the target aneurysm, thereby potentially enhancing the elasticity of the newly formed tissue within the sac. The potential for these materials to fill and encourage cell ingrowth in cerebral saccular aneurysms was assessed in vitro and in vivo using a murine aneurysm model.

## 2 Materials and Methods

### 2.1 Materials

Gelatin (type A, from porcine skin), 1,4-dithiothreitol (DTT), hydrazine hydrate solution (78–82%, iodometric), *N*-(3-(dimethylamino)propyl)-*N*′´-ethylcarbodiimide hydrochloride (EDC, 98%), and alginic acid sodium salt from Macrocystis pyrifera (kelp) were purchased from Sigma-Aldrich (St. Louis, MO, USA). 0.2 M calcium chloride (CaCl_2_) was purchased from Hemonetics (Clinton, PA, USA). Sodium hyaluronate was purchased from Fisher Scientific (Hampton, NH, USA). Divinyl sulfone (stabilized with HQ) was purchased from TCI Chemicals (Tokyo, Japan). Dimethyl 3,3-dithiodipropionate was purchased from Ambeed, Inc. (Arlington Heights, IL, USA). Peptides RADA16 (Ac-RARADADARARADADA-NH_2_) and RADA-SP (Ac-RARADADARARADADAGGRPKPQQFFGLM-NH_2_) were purchased from Peptron (Daejeon, South Korea)

### 2.2 Synthesis and Characterization of Thiolated Gelatin (Gel-SH) and Vinyl Sulfonated Hyaluronic Acid (HA-VS)

Thiolated gelatin (Gel-SH) and vinyl sulfonated hyaluronic acid (HA-VS) were prepared using the methodology described previously.^21^ Briefly, Gel-SH was synthesized from gelatin and 3,3′-dithiobis(propionic hydrazide) (DTPH) which is a reaction product of 3,3′-dithiopropionic acid dimethyl ester with hydrazine hydrate. A solution of 1.0 g of gelatin in 100 mL of distilled water was prepared, and 0.5 g of DTPH was added while stirring. The pH of the solution was adjusted to 4.75 by adding 1 M HCl, followed by the addition of 0.3 g of EDC. After 2 h, the reaction was stopped by adding 1 M NaOH solution to raise the pH to 7.0, then, 2.2 g of DTT was slowly added to the reactor, and the pH was increased to 8.5 using 1 M NaOH. The reaction proceeded for 24 h at room temperature. The pH was adjusted to 3.5 using 1 M HCl, and the acidified product was purified using dialysis membrane tubing (MWCO 12 000) in 0.03 M HCl. The resulting fine powder was obtained through lyophilization.

Hyaluronic acid (HA) and divinyl sulfone (DVS) were used to synthesize HA-VS. A solution containing 0.5 g of HA in 100 mL of 0.02 M NaOH was prepared. Then 1.3215 g of DVS was added to the reactor in an ice bath. The reaction was allowed to proceed for 30 min and then terminated by adjusting the pH to 5.0 using 1 M HCl solution. The product was purified using dialysis membrane tubing (MWCO 12,000) in distilled water at pH 5.3. The fine powder was obtained after lyophilization. The synthesized Gel-SH and HA-VS chemical structures were confirmed by ^1^H-nuclear magnetic resonance (^1^H NMR).

Viscosity and elasticity of 2% alginic acid, 1% HA HA-VS, 2% Gel-SH, 2% Gel-SH + 20% iohexol, and 2% Gel-SH + 20% iohexol in 20 mM HEPES were evaluated at 37 °C using a Vilastic-3 viscoelasticity analyzer (Vilastic Scientific, Austin, TX, USA). For the evaluation, shear rate sweep, measurement sequence, measurement frequency, and integration time were preset as 5 to 80 (1/s), 50 increasing shear rate, 2.0 Hz, and 5 s, respectively.

For the inverted vial gelation test, solutions of HA-VS and Gel-SH were prepared in 20 mM HEPES at concentrations of 1% (w/v) and 2% (w/v), respectively. A 0.5 mL HA-VS solution and a 0.5 mL Gel-SH solution were preheated at 37 °C and injected into a 3.7 mL glass vial using a 25 G needle. The vial containing the mixture was then incubated at 37 °C for 30 s, and the sol–gel phase transition was evaluated using the vial inverting method. The internal structure of the formed gel was examined by cross-sectional images of lyophilized scaffolds using a scanning electron microscope (SEM, JSM 6335F, JEOL, Tokyo, Japan).

A rheometer (AR2000EX, TA Instruments, New Castle, DE, USA) was used for evaluation of the rheological change at 37 °C during the gelation. A 0.5 mL portion of 1% (w/v) HA-VS solution and 0.5 mL of 2% (w/v) Gel-SH solution were injected into the center of the Peltier plate at the same time using separated nozzles. The shear storage modulus (*G*′) and loss modulus (*G*″) were measured at a strain of 5%.

The swelling ratio and degradation profiles of HA-VS/Gel-SH were evaluated in vitro. Solutions of 1% (w/v) HA-VS and 2% (w/v) Gel-SH were prepared in 20 mM HEPES, and 75 μL of each solution was injected into each well of a 96 well plate and then mixed and kept at 37 °C for 1 h. The HA-VS/Gel-SH gels were freeze-dried, washed with distilled (DI) water, and freeze-dried before use. For the swelling ratio, the mass of dried samples was measured and then immersed in Dulbecco’s phosphate-buffered saline (DPBS) at 37 °C for 3 h. The mass of swollen samples was measured for the calculation of the swelling ratio (*S*) using the equation , where *M*s and *M*d are the mass of swollen and dried samples, respectively (*n* = 5).

The degradation profile was evaluated by a change in mass for 4 weeks. The mass of samples (*W*_0_) was measured and then immersed in DPBS for 4 weeks. At time points of 1, 2, 3, and 4 weeks, the samples were pulled out, washed with DPBS and DI water three times for each, and freeze-dried before checking the mass (*W*_1_). The degradation of samples was evaluated by an equation change of mass (%) = ((*W*_1_ – *W*_0_)/*W*_0_) × 100 (*n* = 5).

### 2.3 HA-VS/Gel-SH Gelation with Iohexol

HA-VS was dissolved at 1% (w/v), and Gel-SH was dissolved at 2% (w/v) in 20 mM HEPES separately after being sterilized under UV for 10 min. Iohexol was then dissolved in the Gel-SH solution with various concentrations at 5%, 10%, or 15% (w/v, mass of iohexol/a total volume of HA-VS and Gel-SH mixture solution). 75 μL of each solution HA-VS and Gel-SH+iohexol were injected into each 96 well at 37 °C and then kept in an incubator for 30 min followed by lyophilization. X-ray images of the resulting freeze-dried hydrogels were captured by using an OEC 9800 Plus X-ray machine. In addition, cross-sectional images of the freeze-dried hydrogels were obtained using SEM. Each sample was immersed in liquid nitrogen and sliced using a surgical-grade blade to expose the cross section. Before SEM imaging, the sliced samples were mounted on a sample holder and coated with a 5 nm layer of Pt. The average diameters of the pores were measured from the SEM images using a free photo editing software program Fiji (https://imagej.net/software/fiji/).

### 2.4 Blood-Contacting Test of HA-VS/Gel-SH with Iohexol

Fresh whole ovine blood was obtained for this study through jugular venipuncture, using a sodium citrate-containing tube. The collection and handling of the blood samples followed the guidelines set by the National Institute of Health (NIH) for the care and use of laboratory animals. All animal procedures conducted as part of this study were approved by the Institutional Animal Care and Use Committee (IACUC) at the University of Pittsburgh.

To investigate the gelation behavior of HA-VS/Gel-SH+iohexol in blood, solutions of 1% HA-VS and 2% Gel-SH+iohexol (10%) were prepared following the procedure described in section 2.3. Subsequently, 75 μL of each solution was injected into 50 μL of fresh ovine blood in individual wells of a 96-well plate, maintained at 37 °C. As a comparison, 0.2 M CaCl_2_ (20v/v%, relative to the total blood volume) or 2% (w/v) alginic acid in PBS + 0.2 M CaCl_2_ (20v/v%, relative to the total blood volume) were injected into 50 μL of fresh blood. After injection, the plate was placed in an incubator for 30 min. The samples were then washed with DPBS five times to remove any unattached blood cells, followed by fixation with 2.5% glutaraldehyde at room temperature for 2 h. The samples were subjected to lyophilization after being washed with distilled water. The internal structure of the freeze-dried hydrogels was examined by using cross-sectional SEM images. The number of entrapped or encapsulated red blood cells was counted from the SEM images.

To explore the interaction between blood cells and the scaffolds following gelation, lyophilized HA-VS/Gel-SH+iohexol (10% or 15% of the total mixture volume) and 2% alginic acid+0.2 M CaCl_2_ scaffolds were exposed to fresh ovine blood, and the results were observed by SEM imaging. The lyophilized hydrogels were prepared as described previously and immersed in 5 mL of fresh ovine blood. The blood-containing tubes with the samples added were maintained at 37 °C with gentle rocking for 3 h. Following blood contact, the samples were washed with DPBS five times to remove any unattached blood cells. Subsequently, the samples were fixed with 2.5% glutaraldehyde at room temperature for 2 h, followed by washing with distilled water. The samples were then lyophilized and sectioned by using a surgical blade in liquid nitrogen to examine their surface and cross-sectional morphologies through SEM imaging. The number of entrapped or encapsulated red blood cells was counted from the SEM images.

### 2.5 rSMC Penetration and Proliferation in HA-VS/Gel-SH with Iohexol

The penetration and proliferation of rat aortic smooth muscle cells (rSMCs) within the HA-VS/Gel-SH+iohexol hydrogel were assessed by the in vitro seeding of rSMCs followed by DAPI imaging. Lyophilized HA-VS/Gel-SH+iohexol (10% or 15% of the total mixture volume) and 2% alginic acid+0.2 M CaCl_2_ scaffolds were prepared following the aforementioned method. The samples were sterilized under UV light for 10 min and placed in separate wells of a 96-well plate. rSMCs were cultured in Dulbecco’s modified Eagle medium (DMEM) supplemented with 10% heat-inactivated fetal bovine serum (HI FBS) and 1% penicillin/streptomycin at 37 °C and 5% CO_2_. A volume of 150 μL of rSMCs at a concentration of 5 × 10^6^ cells/mL was added to each well containing the sample. The culture medium was refreshed daily. After 1 and 7 days, the samples were transferred to fresh wells and washed with DPBS. Following fixation with 2.5% glutaraldehyde, the samples were embedded in an optimal cutting temperature (OCT) compound and then sectioned using a microtome-cryostat (CryoStar NX50, Thermo Scientific). The mounted slices were washed with PBS and stained with 4′,6-diamidino-2-phenylindole (DAPI). The images were captured using a Zeiss fluorescence microscope.

### 2.6 In Vitro Cell Response to HA-VS/Gel-SH with Iohexol and RADA-SP

The gelation of HA-VS/Gel-SH with RADA-SP was tested in vitro, and their morphology was observed by SEM imaging. To prepare the gel incorporating RADA-SP, the following steps were taken: first, HA-VS was dissolved at a concentration of 1% (w/v) and Gel-SH was dissolved at a concentration of 2% (w/v) in 1 mL of 20 mM HEPES solution. Both solutions were sterilized under UV for 10 min. Then, iohexol was added to the Gel-SH solution at a concentration of 20% (w/v), and 2 mg of either RADA or RADA-SP was dissolved in 1 mL of the Gel-SH+iohexol solution. Next, 75 μL of each solution (HA-VS and Gel-SH+iohexol+RADA or RADA-SP) were separately injected into individual 96-well plates at a temperature of 37 °C. The plates were incubated for 30 min in an incubator and subsequently subjected to lyophilization. Finally, the cross-sectional morphologies of the resulting gels were observed by using SEM imaging. The average diameters of the pores were measured from the SEM images using a Fiji.

The cell responses to the RADA-SP-incorporated injectable embolic were evaluated and compared with those of RADA and RADA-SP free embolic and RADA-incorporated embolic in vitro. For the evaluation of the effect of RADA-SP, rat vascular endothelial cells (rECs, from aortic tissue) or rSMCs (150 μL of 1 × 10^7^ cells/mL) were seeded on the lyophilized gels in each well of a 96-well plate and incubated for 7 d. The cell medium was changed daily. After incubation, samples were washed in PBS and fixed in 2% paraformaldehyde for 1 h at room temperature, followed by permeabilization and blocking in PBS containing 0.2% Triton X-100, 3% bovine serum albumin, and 10% goat serum for 2 h at room temperature. After blocking, samples were incubated with primary antibody (ab6994, rabbit polyclonal to Von Willebrand Factor, Abcam, Cambridge, UK, 1:300) or (ab5694, rabbit polyclonal to alpha-smooth muscle actin, Abcam, 1:100) overnight at 4 °C, then incubated with the solution containing secondary antibody (A21442, Alexa Fluor 594 chicken antirabbit IgG H&L, Invitrogen, Eugene, Oregon, USA, 1:500) or (A11034, Alexa Fluor 488 goat antirabbit IgG H&L, Invitrogen, 1:250) and DAPI for 2 h at room temperature. Primary and secondary antibodies were diluted in PBS containing 5% goat serum. Stained samples were kept in PBS until imaging. Fluorescent images were acquired using either a Zeiss fluorescence microscope (for VWF) or a Nikon A1 confocal laser scanning microscope (for αSMA). Image analysis was performed using either Zen lite (version 2.3) or Fiji.

### 2.7 In Vivo Studies Using a Mouse Aneurysm Model

All animal experimentation conducted in this study adhered to a protocol approved by the IACUC at the University of Pittsburgh, in accordance with the guidelines set by the NIH. Given that cerebral aneurysm formation and rupture affect women at a 60% higher rate than men,^24,25^ we used C57BL/6 female mice (Charles River) to establish murine carotid aneurysm. Briefly, mice were anesthetized with an isoflurane and oxygen gas mixture, and then microsurgical exposure of the right common carotid artery (RCCA) was performed under sterile conditions using an operating microscope. The RCCA was exposed, and then a latex cuff was placed around the vessel. The RCCA was bathed with porcine pancreatic elastase solution (10 U/1 mL of PBS) for 20 min, and the vessel was occluded distally. A saccular aneurysm formed over the next 3 weeks. For control purposes, sham-operated animals underwent microsurgical exposure to the RCCA, which was then bathed with phosphate-buffered saline rather than elastase solution and no distal occlusion of the artery. A second control group underwent the same procedure but received elastase but no follow-up treatment with an embolic.

Two different components HA-VS/Gel-SH with 10% (w/v) iohexol and HA-VS/Gel-SH with 10% iohexol and 2 mg of RADA-SP were tested in vivo using the murine model (*n* = 7 for each group). Here, a temporary aneurysm clip with a relatively gentle closing force of 69 gf (0.68N; MIZUHO Sugita Titanium temporary miniclip II) was placed at the neck of the aneurysm to prevent retrograde embolization. A 30G needle was then used to inject ∼20 μL of the embolic agent into the aneurysm. The gel mixture was allowed to cross-link for 5 min. The temporary clip was then removed, and the aneurysm was allowed to heal for 3 weeks, after which the carotid arteries that had been embolized or underwent a sham procedure were collected for proinflammatory cytokine assay, histologic evaluation, and immunofluorescence analysis.

### 2.8 Proinflammatory Cytokine Assay

For proinflammatory cytokine expression, to extract proteins from these arteries, a Cell Lysis Buffer from RayBiotech was utilized for their lysis. Subsequently, the isolated protein was applied onto standard mouse cytokine antibody array membranes (RayBiotech’s Mouse Cytokine Array C1000), which enabled the analysis of 96 different cytokines. The membranes underwent steps involving incubating them with the protein sample, removing any unbound proteins through washing and treating them with a detection reagent. The emitted signals resulting from the bound cytokines were visualized by capturing film images. To assess the intensity of these signals, densitometry analysis was performed using ImageJ. Morpheus, software for matrix visualization and analysis (https://software.broadinstitute.org/morpheus) was employed to visualize the data. Additionally, data clustering was carried out by applying the hierarchical clustering algorithm within Morpheus, using the Spearman rank correlation similarity metric (*n* = 3 animals in each group).

### 2.9 Histologic Evaluation

Aneurysm tissues treated with the injectable embolic were collected and preserved in a 30% sucrose solution at 4 °C for 3 days before use. The samples were embedded in O.C.T. compound and cut into 5 μm serial sections. Subsequently, the slides were subjected to hematoxylin and eosin (H&E), trichrome, and picrosirius red staining according to standard procedures to visualize general morphology, connective tissue formation, and cellular infiltration. The images were taken by a slide scanner (MoticEasyScan Pro 6, Motic, Kowloon City, Kowloon, Hong Kong) and processed with Aperio ImageScope (Leica Biosystems, Wetzlar, Germany).

### 2.10 Immunofluorescence Analysis

For evaluation of cell infiltration, tissue sections were stained with markers for CD68 (macrophages), FSP-1 (fibroblast), MECA32 (endothelial cell), αSMA (smooth muscle cell), CD80 (M1-macrophage), and CD206 (M2-macrophage). The samples were double stained with CD68 (ab53444, rat antimouse recombinant antibody, Abcam, 1:100) and FSP-1 (ab197896, rabbit antimouse recombinant antibody, Abcam, 1:125) and also MECA32 (ab27853, rat antimouse recombinant antibody, Abcam, 1:500) and αSMC (ab5694, rabbit antimouse recombinant antibody, Abcam, 1:100). Also, samples were single stained with CD80 (ab254579, rabbit antimouse recombinant antibody, Abcam, 1:50) and CD206 (ab300621, rabbit antimouse antibody, Abcam, 1:50). Alexa Fluor 488 donkey antimouse IgG H&L (A11006, Invitrogen, 1:500), Alexa Fluor 594 donkey antirabbit IgG H&L (A21442, Invitrogen, 1:500), Alexa Fluor 647 donkey antirat IgG H&L (A78947, Invitrogen, 1:500), and Alexa Fluor 647 donkey antirabbit IgG H&L (A31573, Invitrogen, 1:500) were used for secondary antibodies.

Antisubstance P antibody (NC1/34HL, sc-21715, rat monoclonal IgG Substance P antibody, Santa Cruz Biotechnology, Dallas, TX, 1:125) was used for staining the tissue sections to estimate remaining RADA-SP in the treated aneurysm. Alexa Fluor 647 donkey antirat IgG H&L (A78947, Invitrogen, 1:125) was used as a secondary antibody. DAPI Fluoromount-G (0100-20, SouthernBiotech, Birmingham, AL, USA) was used for DAPI staining and mounting. Image analysis was performed using Fiji (*n* = 4 animals in each group).

### 2.11 Statistical Analysis

The *n*-value refers to the number of replicates for each test. Data are presented as mean ± standard deviation (SD). One-way ANOVA along with Tukey’s test was performed, and *p* < 0.05 was considered statistically significant.

## 3 Results

### 3.1 Synthesis and Characterization of HA-VS and Gel-SH and their Gelation with Iohexol

**Figure 1:**
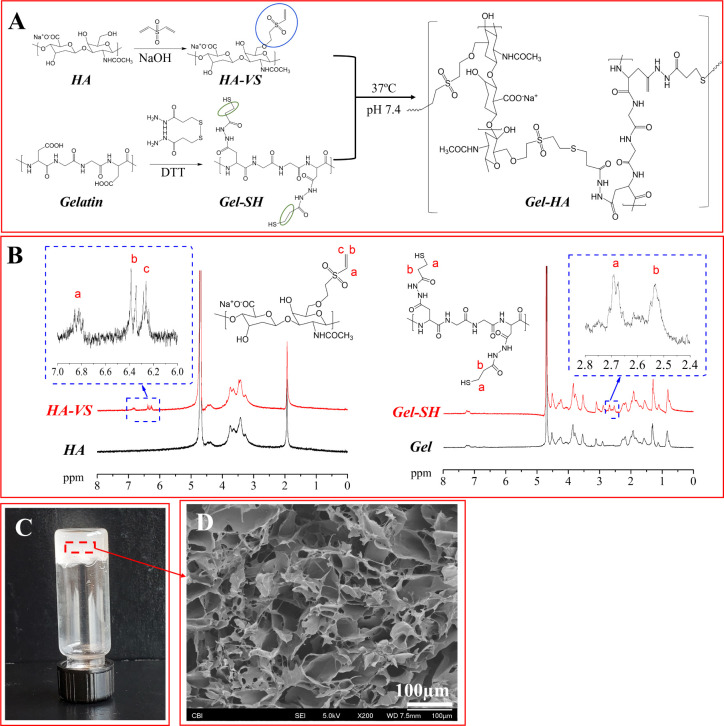
(A) Synthesis of gelatin/hyaluronic acid-based injectable hydrogel; (B) ^1^H NMR of thiolated gelatin (Gel-SH) and vinyl sulfonated hyaluronic acid (HA-VS); and (C) image taken after mixing and (D) SEM of gelatin/hyaluronic acid-based injectable hydrogel.

HA-VS and Gel-SH were successfully synthesized with the expectation of rapid gelation by the Michael Addition reaction when they were mixed at physiological temperature (37 °C) and pH (∼7.4) (Figure 1A). The chemical structure of synthesized HA-VS and Gel-SH were confirmed by ^1^H NMR spectra, which showed new methylene sulfone (−CH_2_CH_2_SO_2_) peaks at 6.25, 6.35, and 6.80 for HA-VS and new side chain methylene (−CH_2_CH_2_SH) peaks at 2.54 and 2.68 ppm for Gel-SH (Figure 1B).^21^ The conversion rate of the repeat unit of HA to HA-VS is 106.5% and that of Gel-SH is 48.1%; therefore, the substitution degree of VS and SH is 106.5% and 48.1%, respectively. The ratio between VS and SH was 1:0.45.

Viscosity and elasticity of 2% alginic acid, 1% HA-VS, 2% Gel-SH, 2% Gel-SH + 10% iohexol, and 2% Gel-SH + 10% iohexol + 2 mg of RADA-SP were evaluated. Their average viscosity with shear rate ranged from 5 to 80 (1/s) were 0.0414 ± 0.0006, 0.033 ± 0.007, 0.0004 ± 0.0002, 0.0024 ± 0.0002, and 0.0028 ± 0.0001 Pa·s, respectively (Supplementary Figure 1A). These viscosities are close to a typical blood viscosity 0.03 Pa·s or lower;^26,27^ therefore, all the prepared solutions can be injected through 5-Fr or smaller catheters. The mixture of 1% HA-VS and 2% Gel-SH became a chemically cross-linked hydrogel at 37 °C and pH 7.4 (Figure 1C), and its lyophilized scaffold showed a porous structure observed by SEM (Figure 1D).

The results of the rheological change and reaction time of the mixture of 1% HA-VS and 2% Gel-SH showed a fast storage modulus increase for 5 min to 88 ± 1 Pa from 49 ± 1 Pa, and then reached 100 Pa within 10 min. The initial storage modulus may be influenced by the premixing of 1% HA-VS and 2% Gel-SH solutions on the Peltier plate before measurement.

The swelling ratio of HA-VS/Gel-SH hydrogel is 271 ± 35%. The mass of HA-VS/Gel-SH scaffolds decreased −24 ± 13% at 1 week and reached −37 ± 16% at 4 weeks (Supplementary Figure 1D).

In consideration of the future potential for angiography-guided transcatheter injection, the FDA-approved water-soluble contrast agent iohexol (Omnipaque) was dissolved in a 2% Gel-SH solution at concentrations of 5%, 10%, or 15% (w/v, mass of iohexol/a total volume of HA-VS and Gel-SH mixture solution). Following the lyophilization process, the resulting chemically cross-linked scaffolds containing iohexol were examined using X-ray and SEM imaging (Supplementary Figure 2). It was observed that all scaffolds with different concentrations of iohexol exhibited radiopacity, with higher concentrations of iohexol yielding greater radiopacity. The SEM images showed that all scaffolds, irrespective of iohexol concentration, displayed porous cross sections. The average pore size (diameter, μm) measured for the iohexol concentration of 5, 10, and 15% was 43 ± 14, 49 ± 12, and 47 ± 11, respectively. However, the scaffolds incorporating 10% and 15% iohexol were deemed better than scaffolds with 5% iohexol considering clear visualization under an X-ray machine.

### 3.2 In Vitro Blood-Contacting of HA-VS/Gel-SH with Iohexol

**Figure 2:**
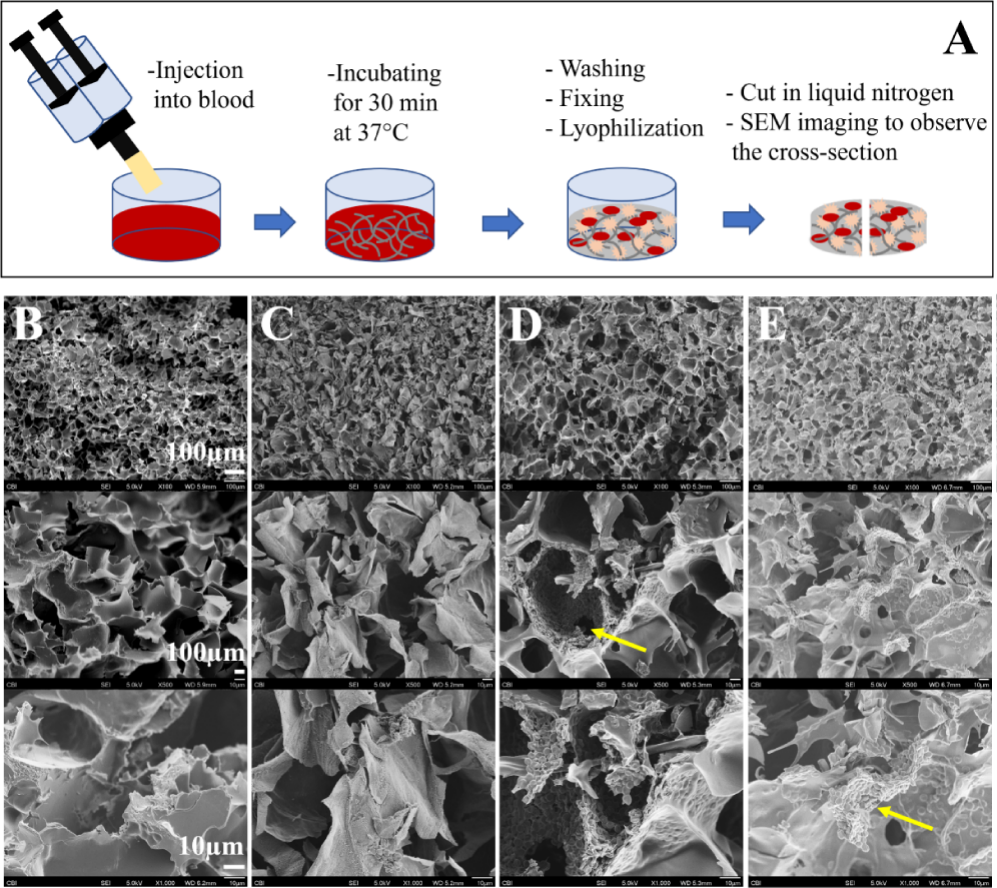
(A) Steps for in vitro injection study with fresh ovine blood; cross-sectional SEM images of injection of (B) 20% 0.2 M CaCl_2_, (C) 2% alginic acid + 20% 0.2 M CaCl_2_, (D) HA-VS/Gel-SH with 10% (w/v) iohexol, or (E) HA-VS/Gel-SH gel with 15% (w/v) iohexol into 50 μL fresh citrated ovine blood (*n* = 4). Arrows indicate blood cell adhesion and incorporation.

SEM images of the cross sections of gels injected into ovine blood are depicted in Figure 2. The HA-VS/Gel-SH gels revealed evident entrapment and encapsulation of blood cells (including red blood cells and platelets) on the porous walls of the gel. In contrast, SEM images of gels formed using 0.2 M CaCl_2_ and 2% alginic acid + 0.2 M CaCl_2_ displayed a less obvious blood cell entrapment in the cross section compared to HA-VS/Gel-SH gels. The number of encapsulated red blood cells was 0, 8 ± 3, 100, and 63 ± 10% for 20% 0.2 M CaCl_2_, 2% alginic acid + 20% 0.2 M CaCl_2_, HA-VS/Gel-SH gel with 10% (w/v) iohexol, and HA-VS/Gel-SH gel with 15% (w/v) iohexol, respectively, when normalized to 20% 0.2 M CaCl_2_ as 0% and HA-VS/Gel-SH gel with 10% (w/v) iohexol as 100%. Overall, the HA-VS/Gel-SH gel incorporating 10% iohexol exhibited the most pronounced attachment of blood cells in the cross section. Distinguishing the formed fibrin from the scaffold was not possible from the images.

**Figure 3:**
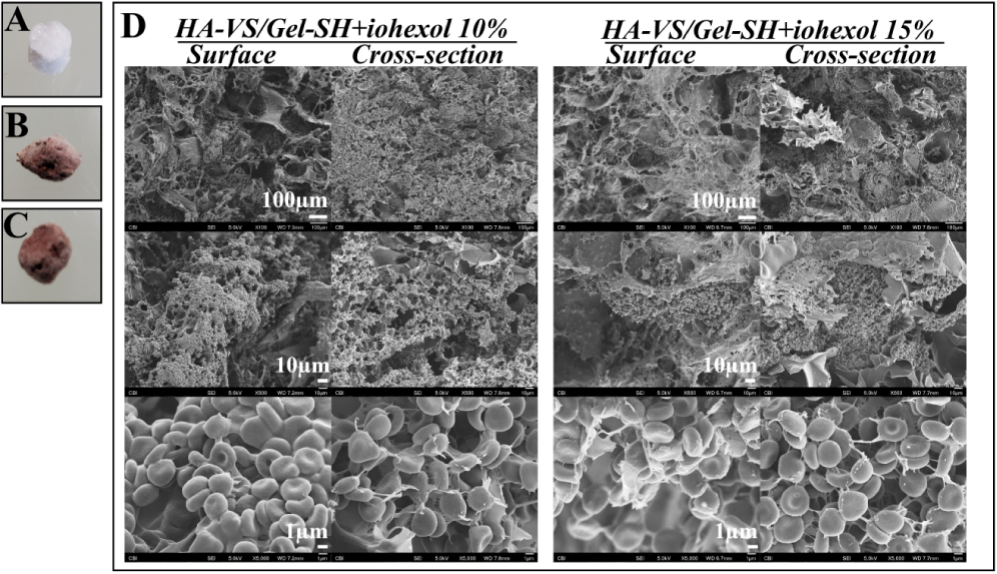
In vitro blood-contacting test where formed HA-VS/Gel-SH gel was lyophilized and contacted ovine blood for 3 h with gentle rocking (*n* = 4): (A) image of freeze-dried HA-VS/Gel-SH with 10% iohexol; (B) freeze-dried HA-VS/Gel-SH with 10% iohexol after blood contact; (C) HA-VS/Gel-SH with 15% iohexol after blood contact; (D) SEM images of surfaces and cross sections from C and D.

From the rocking test with fresh ovine blood, it becomes evident that blood cells were deposited on the surface and penetrated the inner pores of the lyophilized scaffolds (which were formed by the chemically cross-linked HA-VS/Gel-SH+iohexol gels) (Figure 3**)**. The lyophilized scaffold allowed the penetration of blood cells and supported the formation of blood clots within the scaffold. The number of encapsulated red blood cells was 100, 79 ± 6, 67 ± 8, and 72 ± 5% for surface and cross section of HA-VS/Gel-SH gel with 10% (w/v) iohexol, and surface and cross section of HA-VS/Gel-SH gel with 15% (w/v) iohexol, respectively, when normalized to the surface of HA-VS/Gel-SH gel with 10% (w/v) iohexol as 100%. On the other hand, the scaffolds created using 2% alginic acid and 0.2 M CaCl_2_ underwent the same procedure of being in contact with excess blood with gentle rocking for 3 h. However, these scaffolds were fragile and dispersed in the blood, leading to an inability to collect SEM images depicting the blood-contacted 2% alginic acid + 0.2 M CaCl_2_ scaffolds.

### 3.3 In Vitro Cell Response to the HA-VS/Gel-SH

The infiltration and proliferation of cells within the HA-VS/Gel-SH+iohexol gels were assessed for 7 days using rSMCs. The number of cells in the cross section of the gels was estimated by fluorescent images with DAPI staining and the intensity of the DAPI (Figure 3). It was observed that the HA-VS/Gel-SH gel containing 10% iohexol exhibited a significantly higher DAPI intensity on both day 1 and day 7 compared to the HA-VS/Gel-SH gel with 15% iohexol. Furthermore, the DAPI intensity on day 7 was significantly greater than on day 1 for both the 10% and 15% iohexol-containing HA-VS/Gel-SH gels. 2% alginic acid + 20% 0.2 M CaCl_2_ were tested as well, but data could not be collected because of degradation and the fragility of fragments.

**Figure 4:**
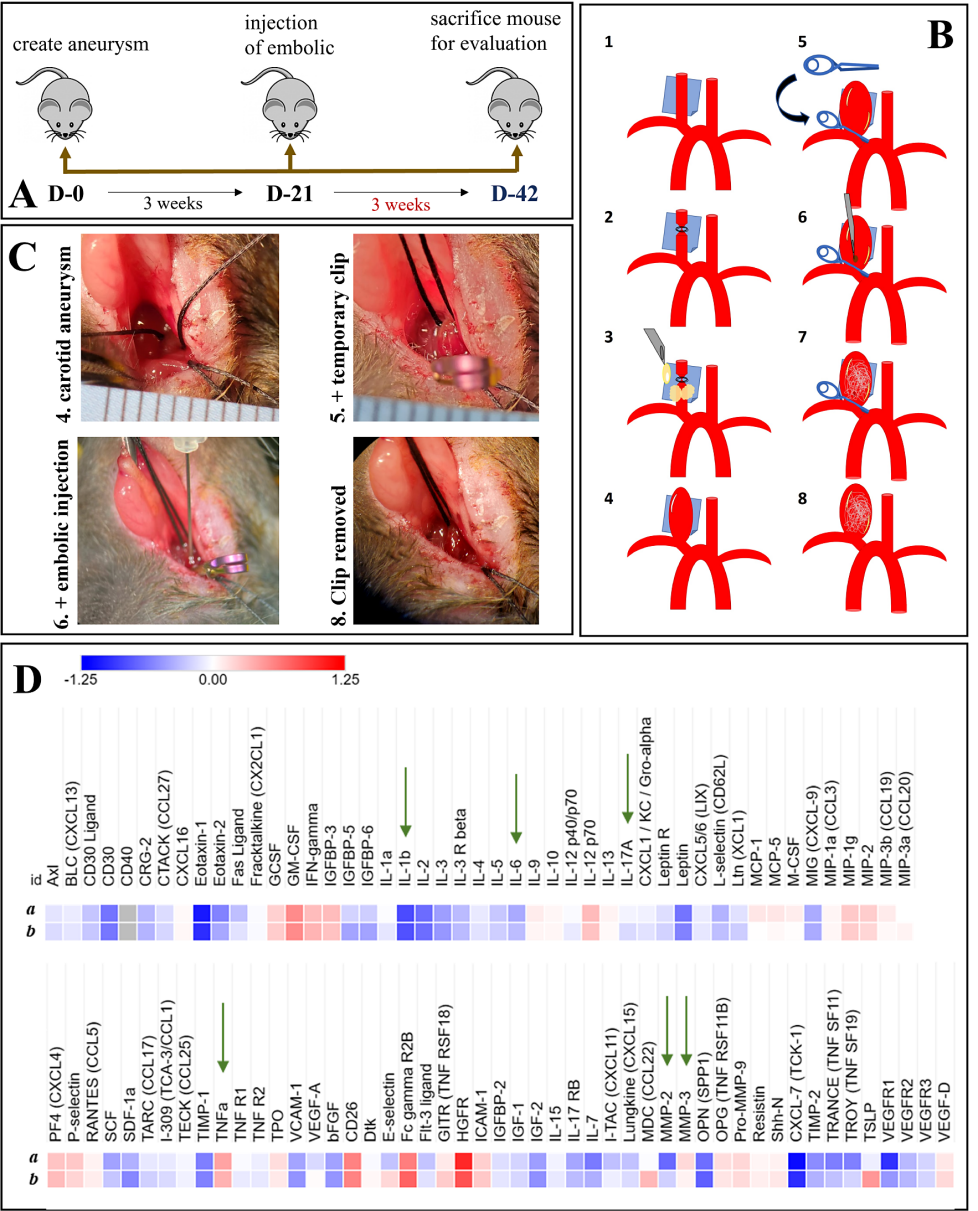
In vivo embolization studies using the mice aneurysm model: (A) diagram of schedule of the animal study; (B) aneurysm creation and embolization steps: 1) murine common carotid artery is exposed and a latex cuff is placed underneath; 2) most distal portion of the artery is ligated using 8–0 nylon suture to create a stump; 3) the artery is incubated with elastase for 20 min; 4) at the end of the incubation period an aneurysm has formed, the latex cuff is removed and the neck incision is closed; 5) 3 weeks later the carotid aneurysm is re-exposed and a temporary clip is placed around the proximal part of the artery-aneurysm complex; 6) embolic is injected through a 30G needle; 7) waiting for 5 min; 8) the temporary clip is removed; and (C) intraoperative photographs of the injection. (D) Pro-inflammatory cytokine production (*n* = 3) after the injection of (a) HA-VS/Gel-SH with 10% iohexol and (b) HA-VS/Gel-SH with 10% iohexol and RADA-SP.

The HA-VS/Gel-SH+iohexol gels containing RADA or RADA-SP exhibited porous cross-sectional images (Figure 4). The average pore size (diameter, μm) measured for the peptide-free, with RADA, or with RADA-SP was 43 ± 14, 35 ± 9, and 29 ± 7, respectively. Among the different types of prepared scaffolds (peptide-free, with RADA, or with RADA-SP) seeded with rECs or rSMCs, the gel incorporating RADA-SP demonstrated significantly higher expression of both von Willebrand factor (vWF) and alpha-smooth muscle actin (αSMA) compared to the peptide-free and with RADA gels. In the case of rEC seeding, the DAPI intensity in the cross section of the RADA-SP added gel was significantly lower, but the ratio of vWF/DAPI was significantly higher compared to other gel compositions (Supplementary Figure 5). When rSMCs were seeded, the gel incorporating RADA-SP displayed significantly higher DAPI intensity, αSMA expression, and αSMA/DAPI ratio compared to the other gel compositions (Figure 6)

### 3.4 In Vivo Injection of HA-VS/Gel-SH with Iohexol and RADA-SP Using Murine Carotid Aneurysm Model

The murine carotid aneurysm model was used for 3 weeks of in vivo assessment of the injectable embolics (Figure 4A–C). From the proinflammatory cytokine array (Figure 4D), there were no significant differences between the two embolic groups in the expression of major cytokines IL-1β, IL-6, IL-17A, TNF-α, MMP-2, and MMP-3, which are mediators implicated in cerebral aneurysm formation and rupture.

**Figure 5:**
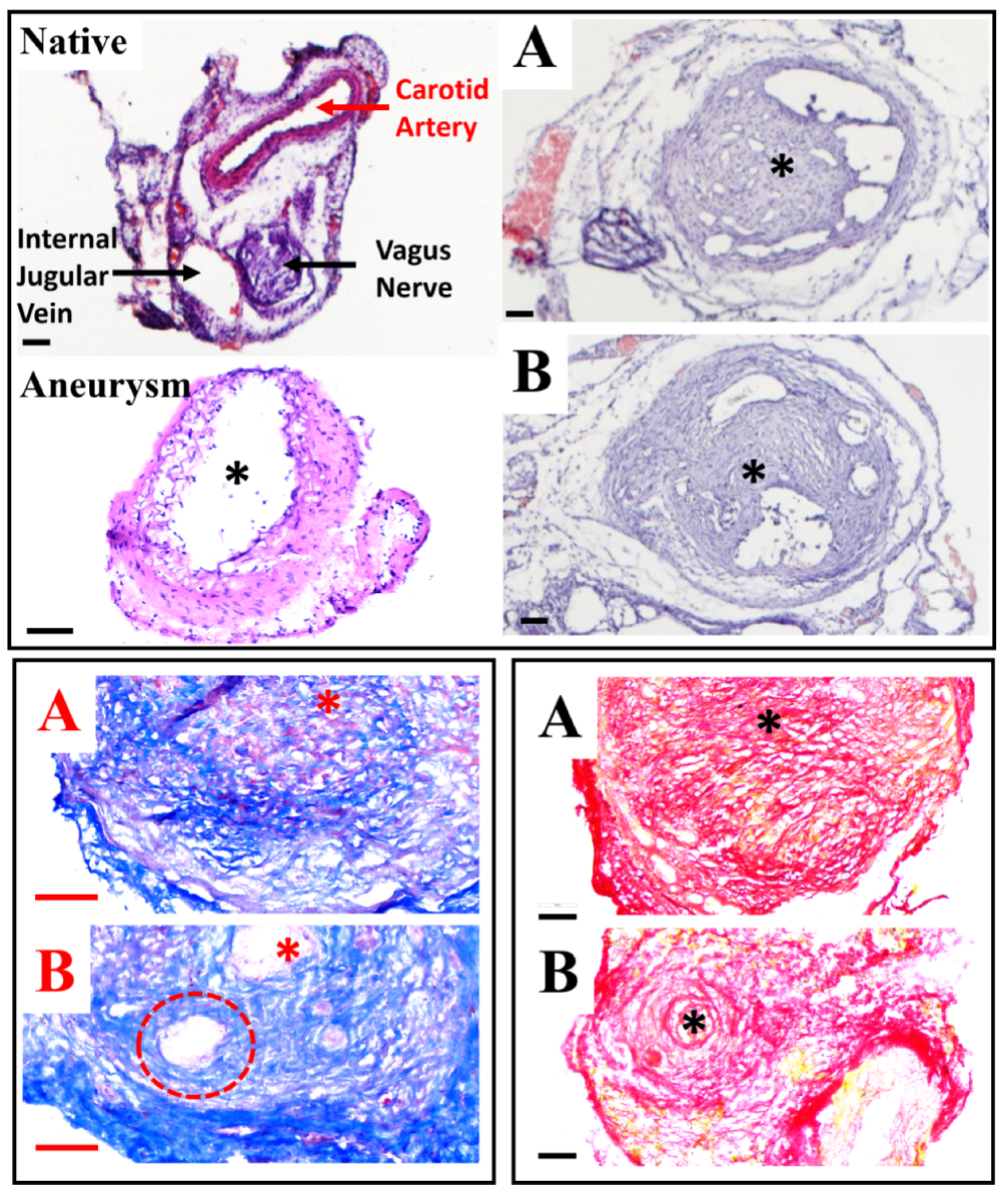
H&E (upper), trichrome (bottom left), and picrosirrus-red (bottom right) images of 3 weeks after the embolization: (A) HA-VS/Gel-SH with 10% iohexol; and (B) HA-VS/Gel-SH with 10% iohexol and RADA-SP (Scale bar: 60 μm, *: middle of aneurysm sac).

Histologic evaluation of H&E, Trichrome, and PicroSirrus-Red showed successful embolization of the aneurysms by the injectable embolic (Figure 5). All of the ECM-mimicking injectable embolic with and without RADA-SP embolized the sac of the aneurysm and allowed cell infiltration.

**Figure 6:**
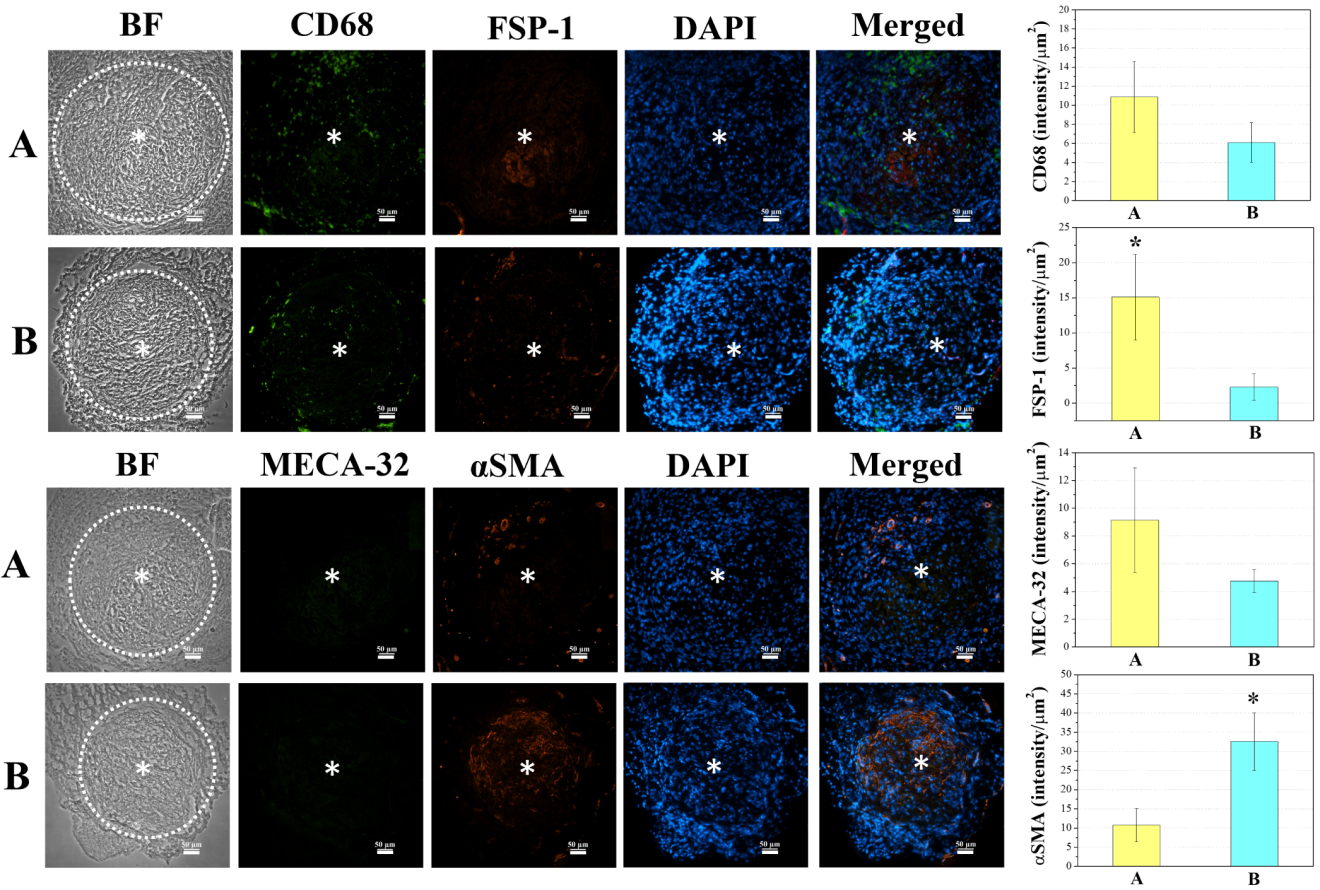
Immunofluorescence (CD68, FSP-1, MECA-32, αSMA, and DAPI) images of 3 weeks after the embolization (*n* = 4): (A) HA-VS/Gel-SH with 10% iohexol, and (B) HA-VS/Gel-SH with 10% iohexol and RADA-SP (scale bar: 100 μm, *: middle of aneurysm sac).

**Figure 7:**
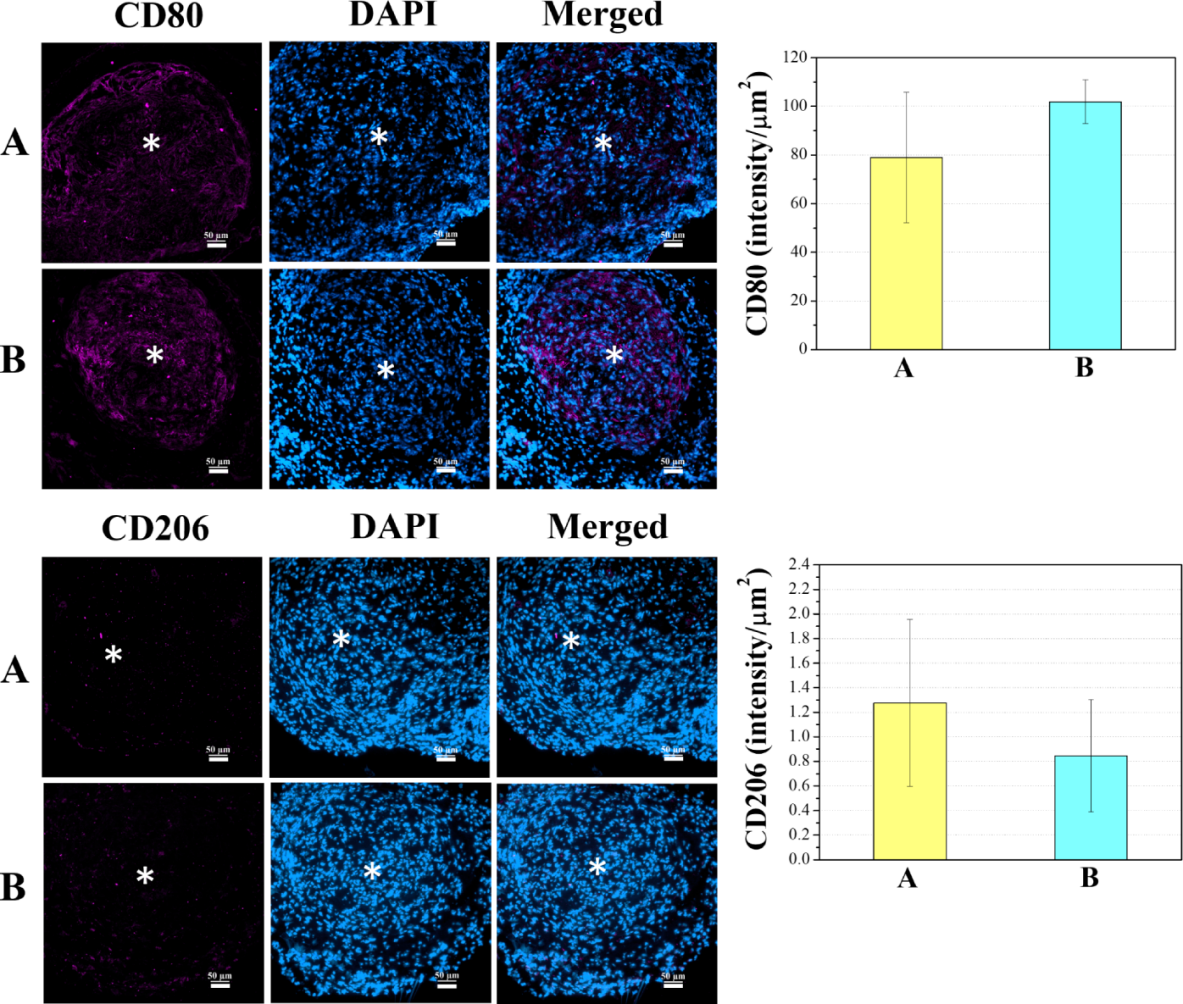
Immunofluorescence (CD80, CD206, and DAPI) images of 3 weeks after the embolization (*n* = 4): (A) HA-VS/Gel-SH with 10% iohexol, and (B) HA-VS/Gel-SH with 10% iohexol and RADA-SP (scale bar: 50 μm, *: middle of aneurysm sac).

To evaluate the infiltration of cells (macrophages, fibroblasts, ECs, SMC, and M1 and M2 macrophages), immunofluorescence staining and imaging were executed with CD68, FSP-1, MECA-32, αSMA, CD80, and CD206 (Figures 6 and 7). The quantification by fluorescence intensity/μm^2^ at the inside of the aneurysm sac showed that HA-VS/Gel-SH with 10% iohexol showed a significantly higher intensity of FSP-1 (15 ± 6) to the embolic with RADA-SP (2.3 ± 1.8). On the other hand, HA-VS/Gel-SH with 10% iohexol and RADA-SP showed a significantly higher intensity of αSMA (32 ± 7) compared to the embolic without RADA-SP (10 ± 4). Both ECM-mimicking embolics showed a higher intensity of M1 macrophage CD80 rather than CD206 in 3 weeks.

The delivered RADA-SP was released within 3 weeks after the injection, and there was no remaining SP in the tissue.

## 4 Discussion

Desirable properties for aneurysm embolization materials would include the promotion of clot formation to effectively exclude the aneurysm sac from the parental artery after embolization and subsequently support the growth of new tissue. Ultimately, the material should be remodeled by the infiltrating cells, allowing for permanent embolization without the presence of a foreign body. This would provide an optimal solution for treating intracranial aneurysms while minimizing potential complications and allowing for easier retreatment, if necessary.

HA and collagen are abundant ECM components broadly found in various tissues. Their cytocompatibility, support for cell adhesion, physical properties, and amenability to purification and modification have made derivatives of HA and collagen common building blocks for biomaterial design in the fields of tissue engineering and drug delivery systems. The use of HA and collagen derivatives (e.g., gelatin) in tissue engineering has shown promising results, as they can mimic critical aspects of the natural ECM and provide structural support. Moreover, their biodegradability ensures that these materials are gradually broken down in the body over time in native remodeling processes without leaving behind permanent foreign substances. Their versatility allows for various modifications and functionalization to tailor their properties for specific applications, such as controlled drug delivery systems.

In this report, we applied a chemically cross-linked hydrogel by synthesizing vinyl sulfonated hyaluronic acid and thiolated gelatin (collagen hydrolyzate functionalized with thiol groups). These components form a hydrogel through the Michael Addition reaction when mixed under physiological conditions. The HA-VS and Gel-SH can be individually delivered to an intracranial saccular aneurysm through two separate channels using a microcatheter connected to a dual-barrel syringe. The delivered two solutions would mix in the sac of the aneurysm, forming a cross-linked ECM-derived matrix while initiating and propagating local thrombus formation.^14,28^ Following in situ gelation, blood can infiltrate the porous embolic material and adhere to its surfaces. This phenomenon was verified through in vitro blood contacting tests conducted using the cross-linked scaffolds. For the test, we chose the alginic acid–based injectable embolic (2% alginic acid +0.2 M CaCl_2_) as a control since the alginic acid is a reasonably close macromolecule to gelatin and hyaluronic acid, as well as that alginic acid-based injectable gel is an FDA-approved embolic and commercialized as Embogel.^29−31^ During 3 h of blood contact, lyophilized hydrogels consisting of 2% alginic acid + 0.2 M CaCl_2_ were observed to degrade in the blood. In contrast, the lyophilized HA-VS/Gel-SH + iohexol gels retained their shape while clot formation occurred. This different behavior between the 2% alginic acid + 0.2 M CaCl_2_ hydrogels and the HA-VS/Gel-SH+iohexol hydrogels can be attributed to differences in the cross-linking mechanism (ionic complexation for alginic acid with calcium ions versus chemical cross-linking for HA-VS/Gel-SH) and the extent of clot formation.

Based on the in vitro data regarding the penetration and proliferation of rSMCs, it was observed that HA-VS/Gel-SH with 10% iohexol had a higher number of cells within the cross section on both days 1 and 7, compared to HA-VS/Gel-SH with 15% iohexol. Iohexol exhibits only low systemic toxicity due to its low chemotoxicity and osmolality. However, a higher iohexol concentration might directly impact cell infiltration and proliferation within the hydrogel by changing the surface chemistry. HA-VS/Gel-SH with 10% iohexol was chosen for the next stage of the experiments.

In endovascular transcatheter embolization, both controlled localized thrombus formation and tissue ingrowth are key factors for successful aneurysm occlusion and healing. In addition to the thrombotic and cell support activity of gelatin, RADA-SP was loaded into the ECM-mimicking injectable embolic with the potential for stimulating improved healing. RADA-SP is a self-assembled peptide conjugated with SP. SP is a neuropeptide released from the peripheral terminals of sensory nerve fibers, and SP functions as both a neurotransmitter and a hormone. Studies have reported that RADA-SP has the ability to enhance the recruitment of endogenous cells into implanted scaffolds and expedite the process of wound healing.^22^ While the effects of RADA-SP on wound healing, including ischemia, bone tissue engineering, skin regeneration, and vascular regeneration, have been extensively studied and established,^22,23,32^ its impact on aneurysm healing has not been investigated to our knowledge.

Self-assembling peptides, such as RADA, possess the ability to form stable β-sheet structures and undergo self-assembly into nanofibers (ranging from 5 to 10 nm) through van der Waals and ionic interactions. RADA-SP has previously exhibited a sustained effect in contrast to free SP by impeding the rapid release of water-soluble SP from the injection site and protecting it from enzymatic digestion and deactivation.^22,23,32^ In an example utilizing a mouse hind limb ischemia model, self-assembled RADA-SP maintained a higher concentration of SP at the target site compared to free SP for 28 days. Resulting in an accelerated wound healing process, attributed to enhanced recruitment of mesenchymal stem cells into the ischemic region.^22^ The sustained release of SP is thus believed to play a vital role in promoting the continuous recruitment of cells and creating a favorable microenvironment for healing. In this regard, it was hypothesized that delivering RADA-SP would aid in healing by facilitating cell recruitment and tissue in-growth into the target aneurysm sac.

For the ECM-mimicking injectable embolic incorporating RADA-SP, given the intricate nature of the physical (e.g., self-assembly of RADA-SP) and chemical (e.g., Michael Addition of HA-VS with Gel-SH) gelation mechanisms involved, it was considered whether HA-VS/Gel-SH with 10% iohexol, in combination with RADA or RADA-SP, displayed a porous interconnected morphology that facilitates cell penetration and metabolite transport. The results demonstrated that the prepared gels containing RADA or RADA-SP exhibited a porous structure. The inclusion of RADA-SP likely introduced variations in surface chemistry, pore size, and structure, which may account for patterns of different cell infiltration into the ECM-mimicking gel.^33^ Furthermore, the gel system incorporating RADA-SP demonstrated notably elevated expression of VWF and αSMA in comparison to other formulations confirmed in vitro. Consequently, the HA-VS/Gel-SH gel with RADA-SP exhibited the potential ability to recruit vSMCs and stimulate angiogenesis. This activity may hold potential for enhancing the healing process of an embolized aneurysm.

IL-1β, TNF-α, and MMPs are mediators that play significant roles in the formation and rupture of cerebral aneurysms.^34^ IL-1β is associated with endothelial dysfunction, while TNF-α is involved in phenotypic modulation and loss of SMCs.^35 −37^ MMPs contribute to phenotypic modulation and SMC loss, macrophage activity, M1/M2 imbalance, leukocyte infiltration, vascular remodeling, and cell death.^38,39^ The expression levels of these mediators were evaluated using a cytokine expression array 3 weeks after implantation, and no significant differences were observed on both of the tested compositions of the injectable embolic. Based on the observation, it appears that integrating RADA-SP into the injectable embolic does not adversely impact cytokine expression associated with aneurysm development and rupture; however, a beneficial effect is also not apparent.

Histological analysis by H&E, trichrome, and picrosirius-red staining revealed that both injectable embolic with different components effectively filled the aneurysm sac and stimulated tissue ingrowth within 3 weeks. When RADA-SP was incorporated into the injectable embolic, potential capillary in-growth was observed. This angiogenesis effect was intended as intracranial aneurysm therapy but their effect on recanalization needs to be evaluated in longer-term animal studies. Immunofluorescent staining with biomarkers revealed that the RADA-SP incorporated injectable embolic showed significantly higher SMC infiltration reflected by αSMA. This result is along the lines of the in vitro αSMA expression data where the RADA-SP showed an effect on SMC proliferation. Nevertheless, given the successful embolization involving macrophages and fibroblast infiltration with the ECM-derived injectable embolic lacking RADA-SP, it is plausible that the injectable embolic without RADA-SP could exhibit superior efficacy in saccular aneurysm embolization and subsequent healing. However, it is worth noting that the hydrogel we developed is capable of incorporating other potential pro-healing molecules (e.g., TGF-β, MCP-1) for cerebral saccular aneurysm treatment. Also, the infiltration of SMCs may induce better elasticity of new tissue in the sac than fibroblast infiltration. Both more extended studies temporally and broader investigation of bioactive factor loading would be logical next steps for this effort.

Several clinical limitations exist with the described approach including: 1) the experimental method used for embolic delivery in vivo, 2) the potential for downstream embolization resulting in cerebral ischemia, and 3) the rapid formation of a low-oxygen environment resulting in local aneurysm wall ischemia and rupture. In this work, we used an open approach and a temporary clip to prevent accidental downstream embolization. This was done to minimize unnecessary animal suffering. However, in clinical practice, such embolization would have to be performed via endovascular balloon-assisted methods. The potential for downstream embolization due to leakage around the balloon and causing a stroke cannot be overstated. In fact, in the CAMEO trial,^10^ a significant percentage of patients treated with Onyx had an intraprocedural complication resulting in permanent neurologic deficit. Another point of concern is that embolic use could result in local aneurysm wall ischemia and early rupture. Hoh et al. described the presence of endothelial cells and capillaries within both human and murine aneurysms.^40^ The function of these capillaries is unknown. However, rapid ischemia within the aneurysm wall could result in cellular apoptosis, a loss of structural integrity, and early rupture.

## 5 Conclusion

Thiolated gelatin (Gel-SH) and vinyl sulfonated hyaluronic acid (HA-VS) were successfully synthesized, and their composition was confirmed using ^1^H NMR. By the rapid reaction of Gel-SH and HA-VS, upon mixing, ECM-derived, chemically cross-linked porous hydrogels were formed, incorporating the contrast agent iohexol. Furthermore, the bioactive, self-assembling peptide RADA-SP could be incorporated without compromising gel formation and structure. The injectable embolic system demonstrated promising potential for treating cerebral saccular aneurysms. In both in vitro and in vivo (murine) models, the embolic material exhibited rapid gelation, forming an in situ scaffold that enhanced thrombus formation and facilitated tissue ingrowth into the aneurysmal sac. Overall, the ECM-mimicking injectable embolic showed the potential for aneurysm treatment; although, further longer-term (over 3 months) in vivo studies may be necessary for a better understanding of their efficacy.

## Supporting Information Available

Supplementary Figure 1: (A) Viscosity test, (B) elasticity evaluation, (C) modulus of HA-VS/Gel-SH, and (D) in vitro degradation profile of HA-VS/Gel-SH with 10% iohexol scaffolds in PBS, Supplementary Figure 2: (A) photo image of HA-VS/Gel-SH gel with 10% (w/v) iohexol after reacted and lyophilized in 96-well plate, (B) X-ray (OEC 9800 Plus), and (C) SEM images of HA-VS/Gel-SH gel with different concentrations of iohexol (*n* = 5), Supplemental Figure 3: in vitro rSMC penetration and proliferation through the HA-VS/Gel-SH with 10% iohexol or 15% iohexol (*n* = 4), the cross section of rSMC seeded injectable gel was stained with DAPI after (A) 1 day or (B) 7 days of seeding, Supplementary Figure 4: SEM images of (A) HA-VS/Gel-SH gel with 10% (w/v) iohexol, Supplementary Figure 5: vWF expression after 7 days from seeding rEC (*n* = 4), Supplementary Figure 6: αSMA expression after 7 days from seeding rSMC (*n* = 4), and Supplementary Figure 7: immunofluorescence (NC1/34HL and DAPI) images of 3 weeks after the embolization (PDF)

## References

[ref1] Khan H.; Sharif M.; Bibi N.; Muhammad N. A Novel Algorithm for the Detection of Cerebral Aneurysm Using Sub-Band Morphological Operation. Eur. Phys. J. Plus 2019, 134 (34), 34 10.1140/epjp/i2019-12432-6.

[ref2] Kim S.; Nowicki K. W.; Ye S.; Jang K.; Elsisy M.; Ibrahim M.; Chun Y.; Gross B. A.; Friedlander R. M.; Wagner W. R. B. Elastomer-Coated Magnesium Alloy Coils for Treating Saccular Cerebrovascular Aneurysms. Biomaterials 2022, 290, 121857 10.1016/j.biomaterials.2022.121857. 36326510

[ref3] Keedy A. An Overview of Intracranial Aneurysms. Mcgill. J. Med. 2006, 9 (2), 141–146. 18523626 PMC2323531

[ref4] Vance A.; Welch B. G. The Utility of Bioactive Coils in the Embolization of Aneurysms. Neurol. Res. 2014, 36 (4), 356–362. 10.1179/1743132814Y.0000000320. 24617937

[ref5] Kim S.; Nowicki K. W.; Gross B. A.; Wagner W. R. Injectable Hydrogels for Vascular Embolization and Cell Delivery: The Potential for Advances in Cerebral Aneurysm Treatment. Biomaterials 2021, 277, 121109 10.1016/j.biomaterials.2021.121109. 34530233

[ref6] Han Y. M.; Lee J. Y.; Choi I. J.; Kim C. G.; Cho S. J.; Lee J. H.; Kim H. B.; Choi J. M. Endoscopic Removal of a Migrated Coil after Embolization of a Splenic Pseudoaneurysm: A Case Report. Clin. Endosc. 2014, 47 (2), 183–187. 10.5946/ce.2014.47.2.183. 24765602 PMC3994262

[ref7] Ogilvy C. S.; Chua M. H.; Fusco M. R.; Reddy A. S.; Thomas A. J. Stratification of Recanalization for Patients with Endovascular Treatment of Intracranial Aneurysms. Neurosurgery 2015, 76 (4), 390–395. 10.1227/NEU.0000000000000651. 25621984 PMC4637161

[ref8] Mascitelli J. R.; Oermann E. K.; Mocco J.; Fifi J. T.; Paramasivam S.; Stapleton C. J.; Patel A. B. Predictors of Success Following Endovascular Retreatment of Intacranial Aneurysms. Interventional Neuroradiology 2015, 21 (4), 426–432. 10.1177/1591019915590070. 26092439 PMC4757327

[ref9] Li X.; Ullah M. W.; Li B.; Chen H. Recent Progress in Advanced Hydrogel-Based Embolic Agents: From Rational Design Strategies to Improved Endovascular Embolization. Adv. Healthc. Mater. 2023, 12, 17 10.1002/adhm.202202787. 36905401

[ref10] Molyneux A. J.; Cekirge S.; Saatci I.; Gál G. Cerebral Aneurysm Multicenter European Onyx (CAMEO) Trial: Results of a Prospective Observational Study in 20 European Centers. AJNR Am. J. Neuroradiol. 2004, 25, 39–51. 14729527 PMC7974182

[ref11] Avery R. K.; Albadawi H.; Akbari M.; Zhang Y. S.; Duggan M. J.; Sahani D. V.; Olsen B. D.; Khademhosseini A.; Oklu R. K. An Injectable Shear-Thinning Biomaterial for Endovascular Embolization. Sci. Transl. Med. 2016, 8 (365), 1–12. 10.1126/scitranslmed.aah5533. 27856795

[ref12] Gaharwar A. K.; Avery R. K.; Assmann A.; Paul A.; McKinley G. H.; Khademhosseini A.; Olsen B. D. Shear-Thinning Nanocomposite Hydrogels for the Treatment of Hemorrhage. ACS Nano 2014, 8 (10), 9833–9842. 10.1021/nn503719n. 25221894 PMC4212795

[ref13] Sheikhi A.; Afewerki S.; Oklu R.; Gaharwar A. K.; Khademhosseini A. Effect of Ionic Strength on Shear-Thinning Nanoclay-Polymer Composite Hydrogels. Biomater. Sci. 2018, 6 (8), 2073–2083. 10.1039/C8BM00469B. 29944151 PMC6085890

[ref14] Zehtabi F.; Montazerian H.; Haghniaz R.; Tseng K.; Mohaghegh N.; Mandal K.; Zamanian B.; Dokmeci M. R.; Akbari M.; Najafabadi A. H.; Kim H.-J.; Khademhosseini A. Sodium Phytate-Incorporated Gelatin-Silicate Nanoplatelet Composites for Enhanced Cohesion and Hemostatic Function of Shear-Thinning Biomaterials. Macromol. Biosci. 1720, 23, 2200333 10.1002/mabi.202200333. PMC9851971 36287084

[ref15] Zehtabi F.; Gangrade A.; Tseng K.; Haghniaz R.; Abbasgholizadeh R.; Montazerian H.; Khorsandi D.; Bahari J.; Ahari A.; Mohaghegh N.; Hosseinzadeh Kouchehbaghi N.; Mandal K.; Mecwan M.; Rashad A.; Roberto de Barros N.; Byun Y.; Ermis M.; Kim H. J.; Khademhosseini A. Injectable Shear-Thinning Hydrogels with Sclerosing and Matrix Metalloproteinase Modulatory Properties for the Treatment of Vascular Malformations. Adv. Funct. Mater. 2023, 33 (51), 2305880 10.1002/adfm.202305880. 38558868 PMC10977963

[ref16] Zhang Z.; Albadawi H.; Fowl R. J.; Altun I.; Salomao M. A.; Jahanyar J.; Chong B. W.; Mayer J. L.; Oklu R. Treatment of Ruptured and Nonruptured Aneurysms Using a Semisolid Iodinated Embolic Agent. Adv. Mater. 2022, 34, 2108266 10.1002/adma.202108266. PMC8917094 34936720

[ref17] Zhang Z.; Albadawi H.; Fowl R. J.; Mayer J. L.; Chong B. W.; Oklu R. Treatment of Ruptured Wide-Necked Aneurysms Using a Microcatheter Injectable Biomaterial. Adv. Mater. 2023, 35 (46), 2305868 10.1002/adma.202305868. PMC10843457 37579579

[ref18] Hozumi T.; Kageyama T.; Ohta S.; Fukuda J.; Ito T. Injectable Hydrogel with Slow Degradability Composed of Gelatin and Hyaluronic Acid Cross-Linked by Schiff’s Base Formation. Biomacromolecules 2018, 19 (2), 288–297. 10.1021/acs.biomac.7b01133. 29284268

[ref19] Luo J. W.; Liu C.; Wu J. H.; Lin L. X.; Fan H. M.; Zhao D. H.; Zhuang Y. Q.; Sun Y. L. In Situ Injectable Hyaluronic Acid/Gelatin Hydrogel for Hemorrhage Control. Mater. Sci. Eng. 2019, 98, 628–634. 10.1016/j.msec.2019.01.034. 30813066

[ref20] Huang J. T.; Chang L. C.; Cheng C. S.; Lin J. J.; Huang S. Y.; Chen S. E. Cytotoxicity Produced by Silicate Nanoplatelets: Study of Cell Death Mechanisms. Toxins 2020, 12 (10), 623 10.3390/toxins12100623. 33003487 PMC7600961

[ref21] Feng Q.; Li Q.; Wen H.; Chen J.; Liang M.; Huang H.; Lan D.; Dong H.; Cao X. Injection and Self-Assembly of Bioinspired Stem Cell-Laden Gelatin/Hyaluronic Acid Hybrid Microgels Promote Cartilage Repair In Vivo. Adv. Funct. Mater. 2019, 29 (50), 1–11. 10.1002/adfm.201906690.

[ref22] Kim J. H.; Jung Y.; Kim B. S.; Kim S. H. Stem Cell Recruitment and Angiogenesis of Neuropeptide Substance P Coupled with Self-Assembling Peptide Nanofiber in a Mouse Hind Limb Ischemia Model. Biomaterials 2013, 34 (6), 1657–1668. 10.1016/j.biomaterials.2012.11.008. 23206876

[ref23] Kim J. E.; Lee J. H.; Kim S. H.; Jung Y. Skin Regeneration with Self-Assembled Peptide Hydrogels Conjugated with Substance P in a Diabetic Rat Model. Tissue Eng. Part A 2018, 24 (1–2), 21–33. 10.1089/ten.tea.2016.0517. 28467735

[ref24] Tada Y.; Makino H.; Furukawa H.; Shimada K.; Wada K.; Liang E. I.; Murakami S.; Kudo M.; Kung D. K.; Hasan D. M.; et al. Roles of Estrogen in the Formation of Intracranial Aneurysms in Ovariectomized Female Mice. Neurosurgery 2014, 75 (6), 690–695. 10.1227/NEU.0000000000000528. 25181430 PMC4399640

[ref25] Fréneau M.; Baron-Menguy C.; Vion A. C.; Loirand G. Why Are Women Predisposed to Intracranial Aneurysm?. Front. Cardiovasc. Med. 2022, 9, 815668 10.3389/fcvm.2022.815668. 35224050 PMC8866977

[ref26] Hund S. J.; Kameneva M. V.; Antaki J. F. A Quasi-Mechanistic Mathematical Representation for Blood Viscosity. Fluids 2017, 2 (1), 10 10.3390/fluids2010010.

[ref27] Sloop G. D. Blood Viscosity: its Role in Cardiovascular Pathophysiology and Hematology; Nova Science Publishers, Inc, 2017.

[ref28] Jin Z.; Fan H.; Osanai T.; Nonoyama T.; Kurokawa T.; Hyodoh H.; Matoba K.; Takeuchi A.; Gong J. P.; Fujimura M. Gluing Blood into Gel by Electrostatic Interaction Using a Water-Soluble Polymer as an Embolic Agent. Proc. Natl. Acad. Sci. U. S. A. 2022, 119 (42), e2206685119 10.1073/pnas.2206685119. 36215508 PMC9586266

[ref29] Barnett B. P.; Hughes A. H.; Lin S.; Arepally A.; Gailloud P. H. In Vitro Assessment of EmboGel and UltraGel Radiopaque Hydrogels for the Endovascular Treatment of Aneurysms. J. Vasc. Interv. Radiol. 2009, 20 (4), 507–512. 10.1016/j.jvir.2009.01.005. 19328428

[ref30] Barnett B. P.; Gailloud P. Assessment of EmboGel-A Selectively Dissolvable Radiopaque Hydrogel for Embolic Applications. J. Vasc. Interv. Radiol. 2011, 22 (2), 203–211. 10.1016/j.jvir.2010.10.010. 21185201

[ref31] Becker T. A.; Kipke D. R.; Brandon T. Calcium Alginate Gel: A Biocompatible and Mechanically Stable Polymer for Endovascular Embolization. J. Biomed. Mater. Res. 2001, 54 (1), 76–86. 10.1002/1097-4636(200101)54:1<76:AID-JBM9>3.0.CO;2-V. 11077405

[ref32] Kim S. H.; Hur W.; Kim J. E.; Min H. J.; Kim S.; Min H. S.; Kim B. K.; Kim S. H.; Choi T. H.; Jung Y. Self-Assembling Peptide Nanofibers Coupled with Neuropeptide Substance P for Bone Tissue Engineering. Tissue Eng. Part A 2015, 21 (7–8), 1237–1246. 10.1089/ten.tea.2014.0472. 25411965 PMC4394880

[ref33] Vu L. T.; Jain G.; Veres B. D.; Rajagopalan P. Cell Migration on Planar and Three-Dimensional Matrices: A Hydrogel-Based Perspective. Tissue Eng. Part B Rev. 2015, 21 (1), 67–74. 10.1089/ten.teb.2013.0782. 25011932 PMC4321976

[ref34] Chalouhi N.; Hoh B. L.; Hasan D. Review of Cerebral Aneurysm Formation, Growth, and Rupture. Stroke 2013, 44 (12), 3613–3622. 10.1161/STROKEAHA.113.002390. 24130141

[ref35] Ali M. S.; Starke R. M.; Jabbour P. M.; Tjoumakaris S. I.; Gonzalez L. F.; Rosenwasser R. H.; Owens G. K.; Koch W. J.; Greig N. H.; Dumont A. S. TNF Induces Phenotypic Modulation in Cerebral Vascular Smooth Muscle Cells: Implications for Cerebral Aneurysm Pathology. J. Cereb. Blood Flow Metab. 2013, 33 (10), 1564–1573. 10.1038/jcbfm.2013.109. 23860374 PMC3790924

[ref36] Lamb F. S.; Choi H.; Miller M. R.; Stark R. J. TNFα and Reactive Oxygen Signaling in Vascular Smooth Muscle Cells in Hypertension and Atherosclerosis. Am. J. Hypertens. 2020, 33 (10), 902–913. 10.1093/ajh/hpaa089. 32498083 PMC7577645

[ref37] Kihara T.; Toriuchi K.; Aoki H.; Kakita H.; Yamada Y.; Aoyama M. Interleukin-1β enhances cell adhesion in human endothelial cells via microRNA-1914–5p suppression. Biochem. Biophys. Rep. 2021, 27, 101046 10.1016/j.bbrep.2021.101046. 34179516 PMC8214032

[ref38] Lu H.; Aikawa M. Many Faces of Matrix Metalloproteinases in Aortic Aneurysms. Arterioscler., Thromb., Vasc. Biol. 2015, 35, 752–754. 10.1161/ATVBAHA.115.305401. 25810296

[ref39] Maguire E. M.; Pearce S. W. A.; Xiao R.; Oo A. Y.; Xiao Q. Matrix Metalloproteinase in Abdominal Aortic Aneurysm and Aortic Dissection. Pharmaceuticals 2019, 12 (3), 118 10.3390/ph12030118. 31390798 PMC6789891

[ref40] Hoh B. L.; Hosaka K.; Downes D. P.; Nowicki K. W.; Wilmer E. N.; Velat G. J.; Scott E. W. Stromal Cell-Derived Factor-1 Promoted Angiogenesis and Inflammatory Cell Infiltration in Aneurysm Walls: Laboratory Investigation. J. Neurosurg. 2014, 120 (1), 73–86. 10.3171/2013.9.JNS122074. 24160472 PMC3877706

