# Supplementary material for: Development of an Injectable, ECM-Derivative Embolic for the Treatment of Cerebral Saccular Aneurysms
Source: Biomacromolecules. 2024 Jul 13;25(8):4879–90. doi: 10.1021/acs.biomac.4c00321 (PMC11323012; doi:10.1021/acs.biomac.4c00321)
Supplement: Supplementary file 1 — bm4c00321_si_001.pdf [file bm4c00321_si_001.pdf]

Supplementary Figures

Title: Development of an injectable, ECM-derivative embolic for the treatment of cerebral saccular aneurysms

Seungil Kim*, Kamil W. Nowicki, Keishi Kohyama, Aditya Mittal, Sangho Ye, Kai Wang, Taro Fujii, Shivbaskar Rajesh, Catherine Cao, Rohit Mantena, Marianna Barbuto, Youngmee Jung, Bradley A. Gross, Robert M. Friedlander, William R. Wagner*

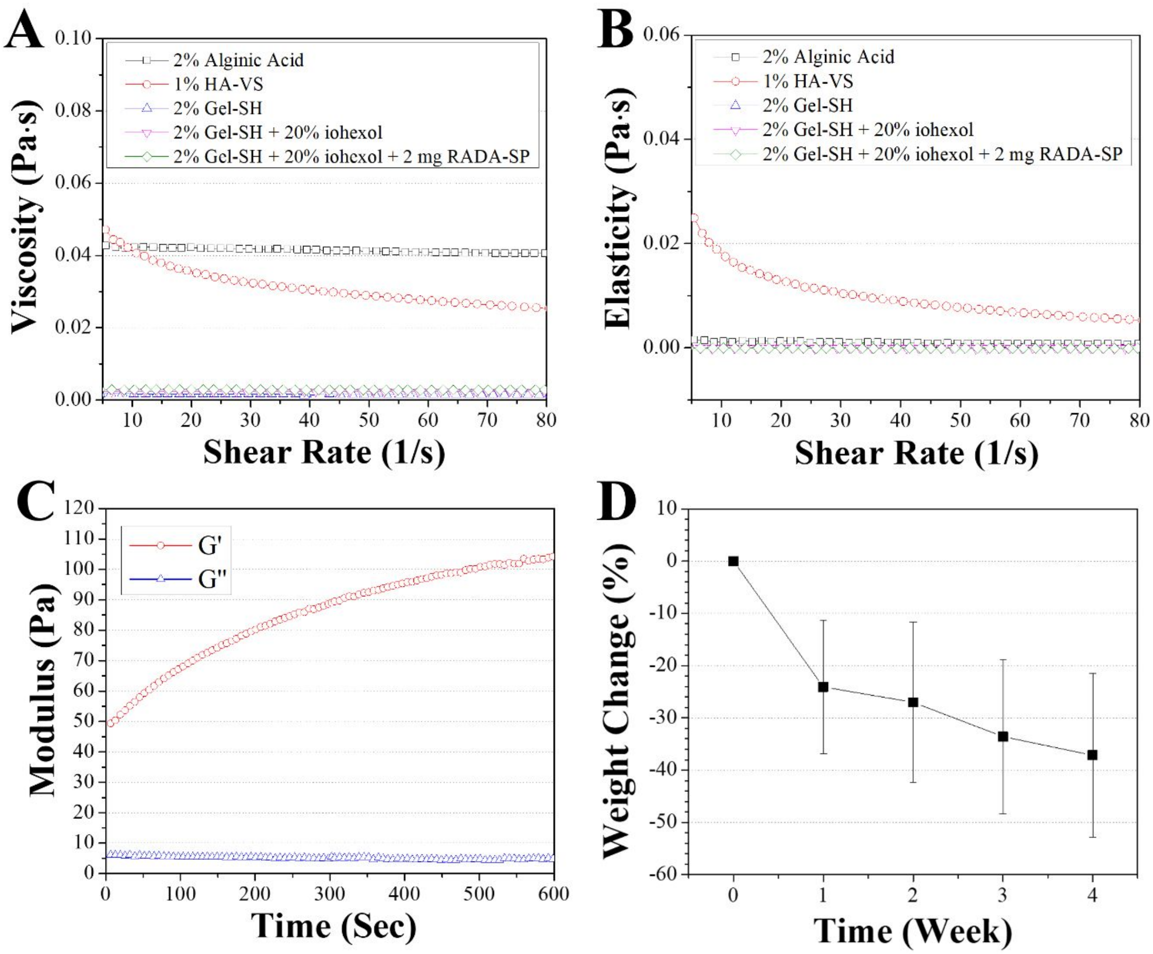

Supplementary Figure 1. (A) Viscosity test, (B) elasticity evaluation, and (C) modulus of HA-VS/Gel-SH. (D) in vitro degradation profile of HA-VS/Gel-SH with 10% iohexol scaffolds in PBS. Weight change versus exposure time was determined for 4 weeks (n =5).

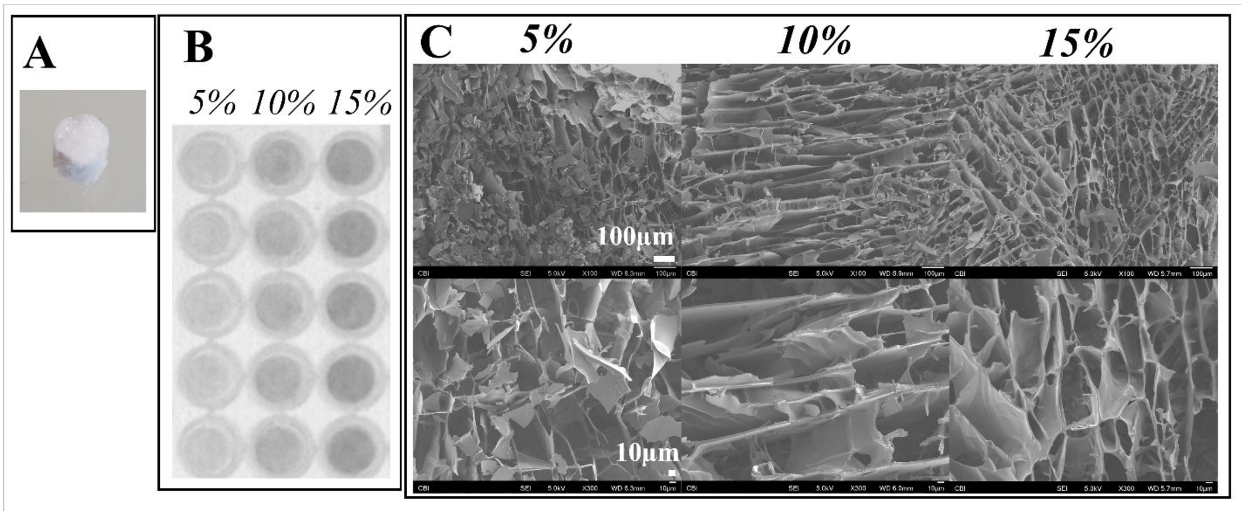

Supplementary Figure 2. (A) Photo image of HA-VS/Gel-SH gel with 10% (w/v) iohexol after reacted and lyophilized in 96-well plate; (B) X-ray (OEC 9800 Plus); and (C) SEM images of HA-VS/Gel-SH gel with different concentrations of iohexol (n=5)

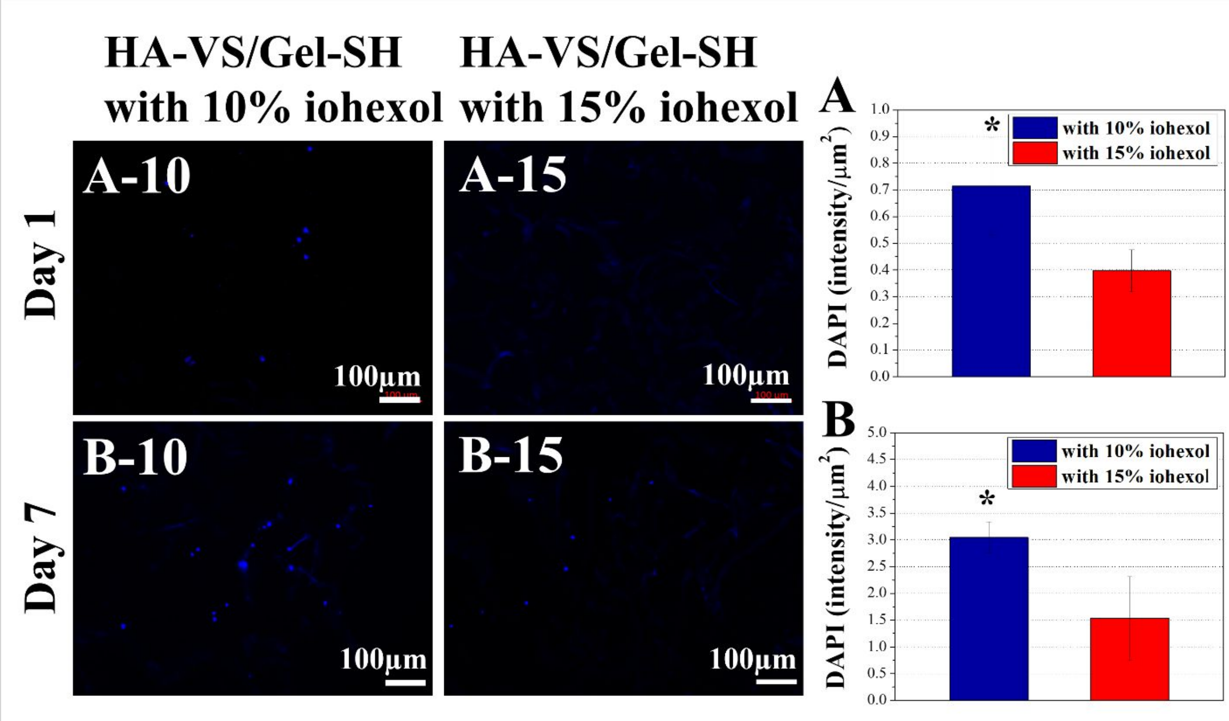

Supplemental Figure 3. In vitro rSMC penetration and proliferation through the HA-VS/Gel-SH with 10% iohexol or 15% iohexol. (n=4) The cross-section of rSMC seeded injectable gel was stained with DAPI after (A) 1 day or (B) 7 days of seeding

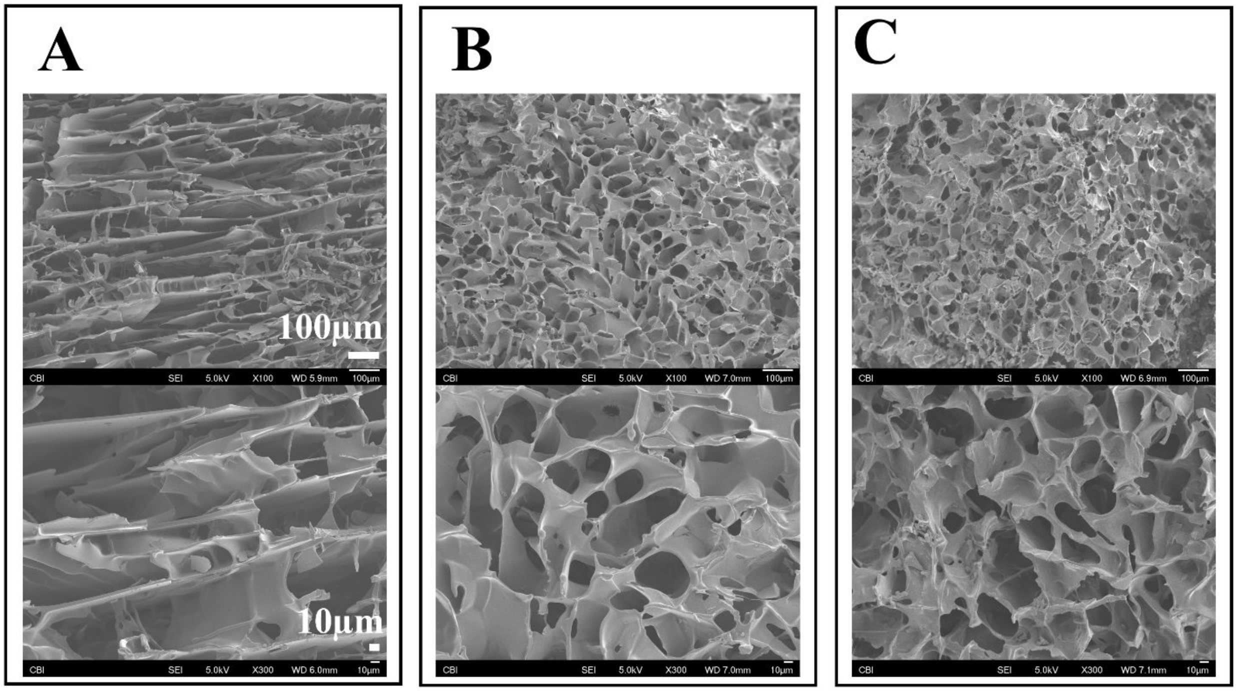

Supplementary Figure 4. SEM images of (A) HA-VS/Gel-SH gel with 10% (w/v) iohexol; (B) HA-VS/Gel-SH gel with 10% (w/v) iohexol and RADA; and (C) HA-VS/Gel-SH gel with 10% (w/v) iohexol and RADA-SP

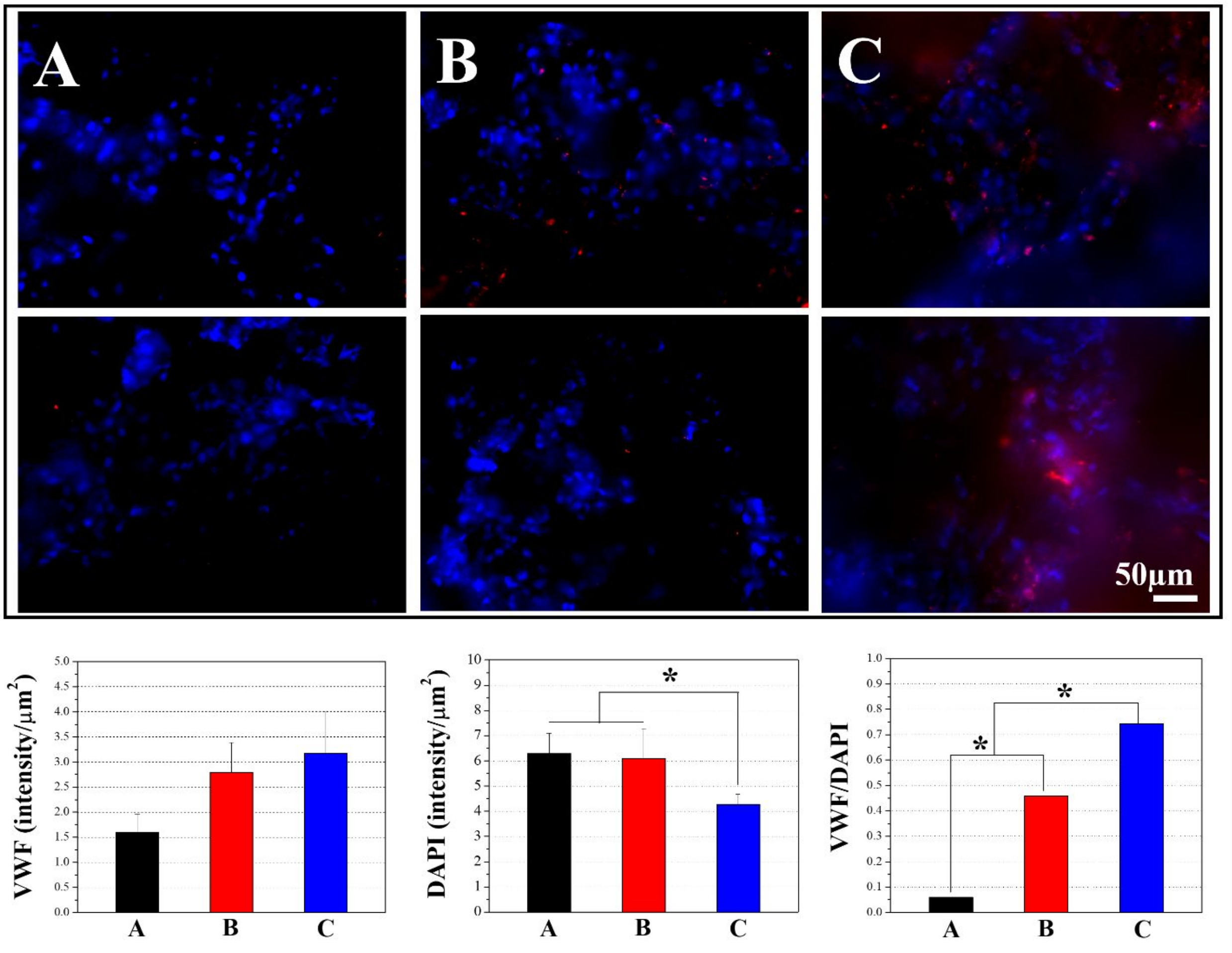

Supplementary Figure 5. vWF expression after 7 days from seeding rEC (n=4): (A) Gel-SH/HA-VS with 10% iohexol, (B) Gel-SH/HA-VS with 10% iohexol and RADA, and (C) Gel-SH/HA-VS with 10% iohexol and RADA-SP

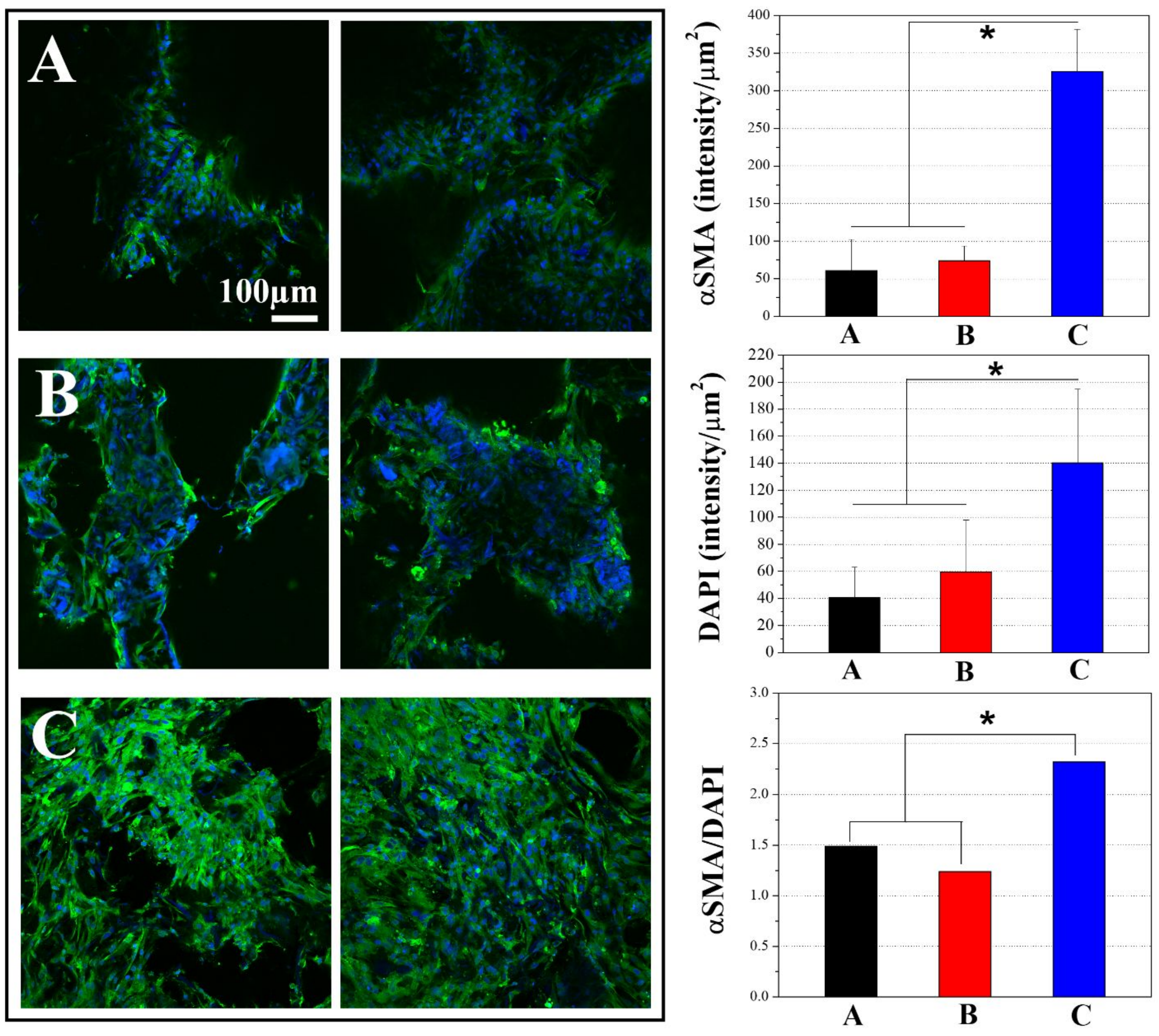

Supplementary Figure 6. αSMA expression after 7 days from seeding rSMC (n=4): (A) Gel-SH/HA-VS with10% iohexol, (B) Gel-SH/HA-VS with 10% iohexol and RADA, and (C) Gel-SH/HA-VS with 10% iohexol and RADA-SP

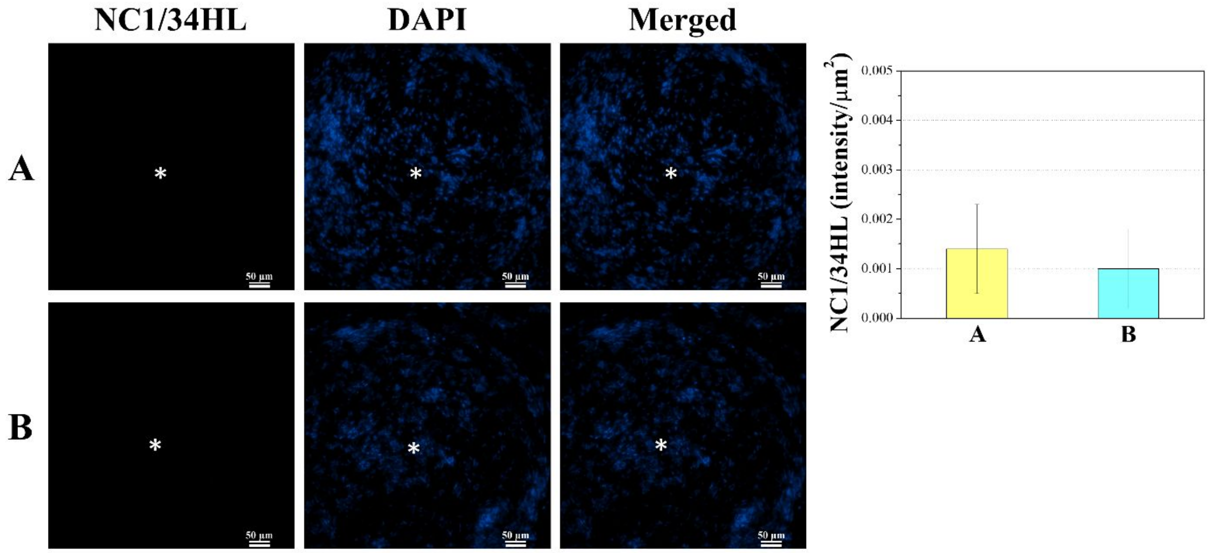

Supplementary Figure 7. Immunofluorescence (NC1/34HL and DAPI) images of 3 weeks after the embolization: (A) HA-VS/Gel-SH with 10% iohexol, and (B) HA-VS/Gel-SH with 10% iohexol and RADA-SP. (Scale bar: 50 µm, ☐: middle of aneurysm sac)
